# LINP1 represses unfolded protein response by directly inhibiting eIF2α phosphorylation to promote cutaneous squamous cell carcinoma

**DOI:** 10.1186/s40164-023-00395-1

**Published:** 2023-03-14

**Authors:** Xiaoting Liang, Jieyu Liu, Xingyuan Liu, Yi Jin, Minna Xu, Zhenyu Han, Ke Wang, Chunting Zhang, Fei Zou, Liang Zhou

**Affiliations:** 1 Department of Toxicology, Guangdong Provincial Key Laboratory of Tropical Disease Research, School of Public Health, Southern Medical University, Guangzhou, China; 2 Institute of Molecular Immunology, School of Laboratory Medicine and Biotechnology, Southern Medical University, Guangzhou, China; 3 Department of Occupational Health and Occupational Medicine, Guangdong Provincial Key Laboratory of Tropical Disease Research, School of Public Health, Southern Medical University, Guangzhou, China

**Keywords:** LINP1, Unfolded protein response, eIF2α, Apoptosis, Cutaneous squamous cell carcinoma

## Abstract

**Background:**

Endoplasmic reticulum stress (ER stress) may destroy endoplasmic reticulum homeostasis (ER homeostasis) and leads to programmable cell death. Unfolded protein response (UPR) originally stimulated by ER stress is critical for the survival of tumor cells through trying to re-establish ER homeostasis as an adaption to harsh microenvironment. However, mechanisms involving key regulators in modulating UPR remain underexplored.

**Methods:**

The expression of LINP1 in cutaneous squamous cell carcinoma (cSCC) tissues and cell lines was assessed. Subsequently, LINP1 was knocked out, knocked down or overexpressed in cSCC cells. CCK-8 assays, colony forming assays, transwell migration assays and invasiveness measurement by matrigel-coated transwell were performed to examine the role of LINP1 in cSCC development through gain-of-function and loss-of-function experiments. Transcriptomic sequencing (RNA-Seq) was conducted and indicated the key downstream signaling events regulated by LINP1 including UPR and apoptosis signaling. Furthermore, the direct interaction between LINP1 and eIF2α to modulate UPR and apoptosis was confirmed by RNA pulldown, RNA immunoprecipitation (RIP), ChIP-qPCR and in vitro phosphorylation assays.

**Results:**

In this study, LncRNA in non-homologous end joining pathway 1 (LINP1) was identified to be one of the top ten highest-expressed LncRNAs in cSCC, the second most common cancer in the world. Functional studies using in vitro and in vivo models revealed that LINP1 functions as an oncogene to promote cell proliferation, colony formation, migration and invasiveness while inhibiting cell apoptosis in cSCC. Transcriptomic sequencing after knockdown of LINP1 indicated LINP1 negatively regulates UPR-related pathways involving key effectors for activating UPR and the apoptosis following the prolonged UPR. Mechanistic study showed LINP1 physically interacts with eIF2α to inhibit its phosphorylation for avoiding unmitigated UPR. Loss of LINP1 followed by enhanced eIF2α phosphorylation led to overactivated UPR and induced DDIT3 expression, contributing to ER stress-induced apoptosis and suppression of cSCC development.

**Conclusions:**

Our findings demonstrate a novel regulatory hierarchy of UPR by demonstrating LINP1 as a critical modulator for eIF2α phosphorylation and a suppressor of UPR-mediated apoptosis, which suggests a novel therapeutic target for cSCC treatment.

**Supplementary Information:**

The online version contains supplementary material available at 10.1186/s40164-023-00395-1.

## Background

Endoplasmic reticulum (ER) is a eukaryotic organelle majorly responsible for proper biosynthesis, folding and transportation of proteins. Correctly folded and assembled proteins will be transported to the cell membrane or released into the extracellular space through the endomembrane system [1]. Protein folding in ER is exquisitely sensitive to internal and external adverse stresses, which lead to the interruption of normal protein folding process, accumulation of unfolded or misfolded proteins and entry into a special condition termed endoplasmic reticulum stress (ER stress) [2–5]. The induction of ER stress starts from the liberation of three ER stress sensors (ATF6, PERK or IRE1α) away from GRP78 (BIP) and could separately trigger downstream signaling events [6] by reprogramming cellular protein translation and gene expression to rescue back to normal ER homeostasis, which is termed unfolded protein response (UPR) [7, 8]. However, prolonged UPR signaling cannot restore homeostasis and be toxic by leading to overwhelming ER stress. Under such conditions, IRE1α and ATF6α branches would be generally attenuated and PERK signaling branch dominates the UPR featured by a series of sequential events including the phosphorylation of eukaryotic translation initiation factor 2 subunit alpha (eIF2α), enhanced translation of activating transcription factor 4 (ATF4), transcriptionally upregulation of DNA damage inducible transcript 3 [DDIT3, also known as C/EBP-homologous protein (CHOP)], activation of death receptor 5 (DR5) and finally execution of apoptosis via caspase-8 involved apoptotic cascade [6, 9]. Tumor cells inevitably face diverse harsh stresses involving hypoxia, nutritional deficiencies and drug toxicity, which would commonly lead to ER stress and the following UPR. Scientists are curious about how tumor cells overcome such endless ER stresses and survive in such harsh microenvironment. Since unmitigated UPR signaling shown by persistent eIF2α phosphorylation is harmful to tumor cells, the survival mechanism shall be tightly related to the modulation of eIF2α phosphorylation.

Currently, the regulation of eIF2α phosphorylation is mainly through two ways: one way is through eIF2α kinases including PERK, while the other way is by regulating the dephosphorylation process of eIF2α. PERK is the only kinase among the three known UPR sensors and responsible for the phosphorylation of eIF2α at Ser51, which is the crucial step to initiate the PERK signaling branch of UPR under ER stress condition [10]. Factors that interfere with or enhance PERK kinase activity can influence eIF2α phosphorylation. For example, ER chaperone GRP78 (BIP) in tumors can associate with PERK to prevent PERK-catalyzed phosphorylation of eIF2α and avoid ER stress-induced apoptosis, thereby promoting malignant phenotype, metastasis and chemotherapy resistance of tumor cells [11]. Endogenous miRNAs can also indirectly regulate the phosphorylation of eIF2α by targeting PERK or GRP78, affecting ER homeostasis in tumor cells. For example, miR-30d, miR-181a and miR-199a-5p targeting GRP78 are downregulated in multiple cancers including colon, prostate and bladder, leading to the upregulation of GRP78 in these cancers to bind more PERK and inhibit its kinase activity for preventing eIF2α hyperphosphorylation-triggered apoptosis [12]. On the other hand, GADD34 transcriptionally regulated by DDIT3 under ER stress conditions could cooperate with protein phosphatase 1 (PP1) to promote eIF2α dephosphorylation [11]. This is an important feedback regulatory mechanism against the ER burden of mRNA translation in response to the ER stress-induced constraint of protein synthesis for avoiding the prolonged phosphorylation of eIF2α to activate downstream apoptotic pathways for survival. However, under persistent ER stress condition, the continuous promotion of eIF2α dephosphorylation by GADD34 will lead to an increase in the level of protein translation load, which will result in the failure of UPR and finally lead to apoptosis instead [11, 13]. Despite the known protein and miRNA regulators, our knowledge about the modulation of eIF2α phosphorylation is still limited.

Long non-coding RNAs (LncRNAs) are a group of noncoding RNAs with length exceeding 200 nucleotides and no protein-coding potential [14]. LncRNAs play diverse roles in chromatin modification, post-transcriptional regulation, genomic imprinting, X chromosome inactivation and miRNA sponge modulation [15, 16] involving pathophysiological processes including carcinogenesis, angiogenesis, muscle development or immune regulation with diverse mechanisms [16, 17]. Recently, accumulating evidences have revealed LncRNAs are also key players during UPR signaling. FOXD3-AS1 could competitively bind to let-7e-5p to regulate RCN1. Silencing FOXD3-AS1 or upregulating let-7e-5p increased the expression profiles of GRP78, CHOP, and ATF4, consequently promoting ER stress-induced apoptosis [18]. MEG3 increased the expression of ER stress-related proteins, including GRP78, IRE1, PERK, ATF6, and CHOP, consequently inhibiting growth and inducing the apoptosis of cancer cells. In addition, MEG3 competitively combines with miR-7-5p or miR-103a-3p to promote ER stress-mediated apoptosis [19]. However, these reports showed that LncRNAs either indirectly modulated ER stress through targeting miRNAs or hardly presented evidence for direct regulation despite the checking of biomarkers for ER stress-induced UPR. Seeing the lack of evidence of direct regulation, we would like to ask whether LncRNAs themselves directly participate and play critical roles in UPR signaling?

Cutaneous squamous cell carcinoma (cSCC), the second most common cancer with an annual incidence exceeding one million worldwide, originates from epidermal keratinocytes [20, 21]. Malignant cSCC is highly aggressive and can actively metastasize to the lymph nodes followed by spreading throughout the body. Patients with cSCC have a high recurrence rate and poor prognosis with a 5 year survival rate of only 22–56% and a 1 year survival rate of only about 50% for patients with recurrence and metastasis [22]. An urgent need to explore the detailed mechanism in cSCC pathogenesis and identify a potential therapeutic target for establishing novel treatment modality of cSCC is necessary. In this study, LncRNA in non-homologous end joining pathway 1 (LINP1) was identified to be significantly upregulated in cSCC tumors and cell lines. Functional studies revealed that LINP1 functions as an oncogene to promote cell proliferation, colony formation, migration and invasiveness but inhibits cell apoptosis in cSCC cells and tumors. Transcriptomic sequencing showed LINP1 may functionally be related with UPR and regulates apoptosis. Loss of LINP1 activates PERK-eIF2α branch-mediated UPR and induces apoptosis in cSCC by upregulating UPR mediator DDIT3 and death receptor DR5. Mechanistic study identified LINP1 directly interacts with eIF2α to repress its phosphorylation for inhibiting PERK-eIF2α branch-mediated UPR signaling and the subsequent apoptosis. Our findings demonstrate that upregulated LINP1 acts as a key regulator to repress UPR-induced apoptosis signaling by inhibiting eIF2α phosphorylation in cSCC, which finally contributes to the development of cSCC.

## Materials and methods

### Patient samples

This study was approved by the Institutional Review Board of Shanghai Outdo Biotech Co. Ltd. (Shanghai, China), all patients provided written informed consent for the use of surgical samples. The tissue array (HSkiC100PT01) that included 57 cSCC specimens and 7 normal cutaneous specimens was purchased from Shanghai Outdo Biotech Co. Ltd. Fresh samples obtained during surgery were immediately frozen in liquid nitrogen for subsequent total RNA and protein extractions and paraffin embedding. Tumors were classified according to the SCC Broders Pathological Classification [43]: stage I (well differentiated) with 75–100% differentiated cells, stage II (moderately differentiated) with 50–75% differentiated cells and stage III and IV (poorly differentiated) with 0–50% differentiated cells.

### Animal studies

This study was approved by the Institutional Animal Care and Use Committee (IACUC) of Nanfang Hospital affiliated with the Southern Medical University (Approval code L2018024). All experiments were performed in accordance with the guidelines of the Asian Federation of Laboratory Animal Science Associations (AFLAS) and the National Regulations for the Administration of Affairs Concerning Experimental Animals (8 January 2011). Details of Mouse transportation, housing, and breeding were conducted according to the recommendations of “The use of non-human animals in research.” Methods of euthanasia accord with international conventions or refer to current guidelines of the AVMA Panel on Euthanasia. During sample collections, mice were euthanized by cervical dislocation to prevent suffering.

### Cell lines

cSCC lines HSC-1 (Male, HonSun Biological Co. Ltd.), A431 (Female, CellCook Biotech Co. Ltd.) and the human benign epidermal keratinocyte cell line HaCaT (Male, CellCook Biotech Co. Ltd.) were grown in Dulbecco’s modified Eagle medium (DMEM, Life Technologies) supplemented with 10% fetal bovine serum (ExCell Bio, FSP500) and maintained at 37 °C with 5% CO_2_ in a humidified atmosphere. The authentication information of all the cell lines used in this study were listed in Additional file 1: Fig. S7–S9. All the cell lines have been tested and shown negative for mycoplasma contamination.

### Isolation of primary human keratinocytes

Fresh foreskin tissue was placed in a container containing 1 × PBS (pH 7.4) on ice. The tissues were cleaned twice with 1 × PBS containing penicillin (500 units/mL) and streptomycin (50 μg/mL) and disinfected with 75% ethanol. Blood vessels and fatty tissue from the foreskin were removed using scissors and tweezers and the tissues were incubated with 3 mL 1 × dispase (1.2–2.4 U/mL) at 4 °C for 12–18 h. The epidermis was detached and placed into 2–3 mL of 0.05% trypsin. It was then incubated at 37 °C for 15 min in a 50 mL falcon tube. Digestion was terminated with 2 mL DMEM containing 10% FBS. After passing 200 mesh cell filter, the cells were transferred to centrifuge tube 400 g for 10 min. The supernatant was discarded and the cells were washed and precipitate with PBS for 2 times. Then, the cells were resuspended with 2 mL primary keratinocytes special medium and cultured for the following assays.

### RNA isolation and qPCR

The total RNA was extracted from cells or tissues by Trizol reagent (TransGen Biotech Co., Ltd.) according to the manufacturer’s instructions. And subsequently reversely transcribed into cDNA using TransScript Uni All-in-One First-Strand cDNA Synthesis SuperMix for qPCR (One-Step gDNA Removal) (TransGen Biotech, AU341). mRNA expression analysis was performed using PerfectStart Green qPCR SuperMix (TransGen Biotech, AQ601) on a LightCycler 96 Detection System (Roche) using GAPDH for normalization. Primers used in this study are listed in Additional file 6: Table S5. 2^−ΔΔCt^ illustrated the fold changes in the target gene expression between the experimental group and the control group. All the qPCR experiments were repeated 3 times.

### Transcriptomic sequencing analysis

Transcriptomic sequencing was performed at RiboBio Co., Ltd. using the Illumina HiSeq 2500 instrument. RNA-Seq data was aligned to the reference genome (human assembly GRCh37/hg19) using Tophat2 (http://ccb.jhu.edu/software/tophat/index.shtml). HTSeq (http://www-huber.embl.de/HTSeq) was then applied on the aligned data set to determine differentially expressed genes with a “significant” status. The Gene Ontology and KEGG analyses of the differentially expressed genes were performed using DAVID (https://david.ncifcrf.gov/). For Clustering analysis of public GEO (http://www.ncbi.nlm.nih.gov/geo, accession GSE139505), z-score transformation of the normalized expression of top 10 upregulated and downregulated genes were calculated as previous described [39]. The output z values were used to generate a heat map.

### In situ* hybridization (ISH) and Fluorescence *in situ* hybridization (FISH)*

Antisense single-stranded DNA probe (Additional file 6: Table S5) was synthesized and end-labelled with digoxigenin (DIG) (Roche). ISH or FISH was performed in formalin-fixed paraffin-embedded melanoma sections or slides covered with cultured melanoma cells. The pre-hybridization, hybridization, anti-DIG-HRP IgG fraction monoclonal (Jackson, 200-032-156) incubation (1:200) and stained with DAB (Servicebio, G1211) was performed as described in previous studies. Stained ISH or FISH sections were imaged with a ZEISS Axio Vert.A1 microscope and at least 10 representative images were collected for statistical analysis. The ISH or FISH staining was performed “blind” with respect to the different treatments [44]. Co-localization of LINP1 with eIF2α in cSCC cells was detected using FISH for LINP1 and immunofluorescence staining for eIF2α and observed by confocal microscope.

### Cell transfection

cSCC cells in exponential growth phase were used for cell transfection. Before transfection, the cells were cultured in 60 or 100 mm dishes with complete medium for 24 h until they were 90% confluent. Transient transfection of cells with siRNA oligos or DNA plasmids was performed with TransIntro EL Transfection Reagent (TransGen Biotech, FT201) as suggested by the manufacturer. Then, the cells were cultured with DMEM medium following the instructions. 36 h after transfection, cell lysates were subjected to western blot and total RNA were extracted and purified from cSCC cells using the Trizol reagent according to the manufacturer’s instructions.

### HPLC–MS analysis

A 20 μg sample of immunoprecipitated protein mix was separated by sodium dodecyl sulfate-polyacrylamide gel electrophoresis (SDS-PAGE) and stained with Coomassie brilliant blue R250 and then processed with Trypsin Profile IGD Kit (Sigma, PP0100). The resulting digest was treated with ZipTip C18 (Merck Millipore, ZTC18S096) then subjected to analysis by Thermo Fisher Scientific orbitrap fusion LC-MS/MS in positive ion, linear, delayed-extraction mode. Calibration was carried out using a standard peptide mixture. The mass spectra were subjected to sequence database search with Proteome Discoverer v2.1 software (Thermo Scientific).

### Generation of LINP1 knockout cell strains

For LINP1 knockout, the single guide RNAs (sgRNA) were designed using the online CRISPR design tool (CRISPOR, http://crispor.tefor.net/) [45]. A ranked list of sgRNAs was generated with specificity and efficiency scores. Two sgRNAs were selected which flank the genomic locus of LINP1. All sgRNAs were accessed using the online, off-target searching tool (Cas-OFFinder; http://www.rgenome.net/cas-offinder). The pair of oligos was annealed and ligated to Bbs I-digested pSpCas9BB)-2A-Puro (PX459) V2.0 (Addgene plasmid #62988) respectively [46]. Such two pX459 plasmids containing each target sgRNA sequences were cotransfected into cells with Lipofectamine 2000 (Thermo Fisher Scientific). After isolation of clonal cell lines by dilution, 100 cells were seeded into each well of a 96-well plate. After the selection of single colonies, colonies with genomic knockout of LINP1 were determined by Sanger sequencing with isolated genomic DNA and LINP1 expression levels in each clone were validated by qPCR. The sgRNAs and primers for CRISPR design and genomic validation are shown in Additional file 6: Table S5.

### ChIP-qPCR analysis

The chromatin immunoprecipitation (ChIP) procedure was performed using the EZ ChIP^™^ Chromatin Immunoprecipitation kit manual (Merck Millipore, Cat. no. 17-371) following the manufacturer’s instructions. 5 μg antibodies against ATF4 (CST) or isotype IgG (Merck Millipore) used as a negative control were added and the complex co-precipitates are captured by Protein G magnetic beads. Genomic DNA pellets were purified using phenol chloroform extraction and ethanol precipitation, and then resuspended in 20 μl water, at which point it is ready for PCR. Relative enrichment was calculated as the amount of amplified DNA normalized to input and relative to values obtained after normal IgG immunoprecipitation, which were set as 1. Primers used are listed in Additional file 6: Table S5.

### Immunoblotting and IHC assays

Total cell protein extracts were prepared and assayed by western blot as previously described [44]. The following primary antibodies and dilutions were used: eIF2α (Santa Cruz Biotechnology, sc-133132, 1:2000), p-eIF2α (Cell Signaling Technology, #3398, 1:2000) GRP78 (Santa Cruz Biotechnology, sc-13539, 1:2000), XBP1 (Santa Cruz Biotechnology, sc-8015, 1:2000), ATF4 (Cell Signaling Technology, #11815, 1:2000), DDIT3 (Santa Cruz Biotechnology, sc-7351, 1:2000), DR5 (Santa Cruz Biotechnology, sc-166624, 1:2000), Caspase-8 (Santa Cruz Biotechnology, sc-81656, 1:2000), Caspase-3 (Santa Cruz Biotechnology, sc-56053, 1:2000), Caspase-7 (Santa Cruz Biotechnology, sc-56063, 1:2000) and GAPDH (Santa Cruz Biotechnology, sc-25778,1:5000). The following secondary antibodies were also used: anti-mouse IgG-horseradish peroxidase (HRP), anti-rabbit IgG-HRP, and anti-goat IgG-HRP (Santa Cruz Biotechnology). Bound antibodies were visualized with the Luminata Forte Western HRP substrate (Millipore).

Xenograft tumors were formalin-fixed and paraffin-embedded and sectioned for IHC staining. The following antibodies were used: GRP78 (Santa Cruz Biotechnology, sc-13539, 1:100), XBP1 (Santa Cruz Biotechnology, sc-8015, 1:100), DDIT3 (Santa Cruz Biotechnology, sc-7351, 1:100) and DR5 (Santa Cruz Biotechnology, sc-166624, 1:100). Stained sections were imaged using BX53 microscope (Olympus) to get representative images for statistical analysis.

### Cell proliferation and colony forming assays

An equal number of cells (5000 per well) transfected with siRNAs were plated in 96-well plates using 5 wells for technical replicates. After 0, 24, 48, and 72 h, the cells were incubated with 10 μL CCK-8 solution in cell counting kit (TransGen Biotech, FP101) at 37 °C for 1.5 h. The incubated plate was then placed into a microplate reader in order to determine the optical density (OD) value at the wavelength of 450 nm. For the colony forming assay, transfected cells were incubated in 6-well plates with 1500 cells per well, which were maintained in DMEM. Medium was replaced 2 times. The cells were cultured for 10 days before they were washed twice with PBS, fixed in 4% paraformaldehyde for 30 min and stained with 0.1% crystal violet. Visible colonies were photographed and counted.

### Apoptosis assay

Cells were seeded on a 60 mm dish and transfected with siRNAs and cultured for 36 h. Apoptotic cells were quantitated using the TransDetect Annexin V/PI cell apoptosis detection kit (TransGen Biotech) according to the manufacturer’s instructions. Briefly, the cells were harvested and washed with phosphate buffer saline (PBS). Then, cells were resuspended in binding buffer and incubated with Annexin V/propidium iodide (PI) for 15 min at room temperature in dark before analysis using a flow cytometer.

### Transwell assay

In vitro migration assay was performed using transwell chambers. 1 × 10^5^ cells transfected with siNC or siLINP1 were seeded into the 8 μm upper chambers of 12-well plates (Merk Millipore) in serum-free DMEM. During culture at 37 °C for 48 h, the cells in the upper chambers were attracted by the DMEM medium supplemented with 10% fetal bovine serum in the lower chamber. The chambers were washed with PBS twice and fixed with 3.7% formaldehyde. Cells were permeabilized using 100% methanol at room temperature and stained with 0.1% crystal violet. After scraping the cells remained in the wells off with cotton swabs, images were captured from each membrane and the number of migratory cells was counted under a microscope.

### Matrigel invasiveness assay

For the assessment of invasive ability, Cells transfected with siNC or siLINP1 were concentrated to 2 × 10^5^ cells in cell suspension and then added to the upper chamber (Merck Millipore) coated with Matrigel for the invasion assay. Other treatments were performed as in the migration assay.

### RNA-pulldown assay

Biotin-labeled RNAs were transcribed in vitro using RNA max-T7 biotin-labeled transcription kit (Ribo Biotechnology Co., Ltd.). The above RNAs were denatured at 90 °C for 2 min and then renatured with RNA structure buffer at RT for 20 min. A431 cell pellets (5 × 10^6^).were resuspended in 1 ml RIP buffer (150 mM KCl, 25 mM Tris pH 7.4, 0.5 mM DTT, 0.5% NP40, 1 mM phenylmethyl sulfonyl fluoride and 1 × PIC) and sonicated with 10 cycles (30 s interval, 30 s sonication). After centrifugation at 13,000 rpm for 10 min, total cell lysate was mixed with 3 μg of renatured RNA respectively and incubated with rotation for 1 h at RT. Each pull-down reaction were mixed with thirty microlitres of washed streptavidin agarose beads (Life Technologies) at RT for 1 h. After washing thoroughly three times, the RNA–protein binding mixture was boiled in SDS buffer and the eluted proteins were detected by western blot or mass spectrometry.

### RNA immunoprecipitation (RIP) assay

Cells were cross-linked with 1% formaldehyde and collected for lysis by radioimmunoprecipitation assay (RIPA) buffer (50 mM Tris pH 7.4, 150 mM NaCl, 1 mM EDTA, 0.1% SDS, 1% NP-40 and 0.5% sodium deoxycholate, 0.5 mM DTT, 1 mM phenylmethyl sulfonyl fluoride, 1 × Proteinase inhibitor cocktail and 1% RNase Out). The lysate was incubated with eIF2α antibody or normal IgG control for overnight. The RNA/protein complex was recovered with protein G Dynabeads and washed with RIPA buffer several times. After reverse cross-link with proteinase K at 45 °C for 45 min, RNA was recovered with Trizol and analysed by RT–qPCR.

### Xenograft mouse model

4–5 week-old female NCG mice (NOD/ShiLtJGpt-Prkdc^*em26Cd52*^*Il2rg*^*em26Cd22*^*/*Gpt), purchased from the Guangdong Gempharmatech Biotechnology Co., Ltd., were used for establishing xenograft model. Equal number of NCG mice were assigned to siNC and siLINP1 groups respectively. 0.2 mL of above cell suspension that contained 4 × 10^6^ cells were subcutaneously implanted into the left and right flanks of nude mice. The tumor diameters were measured and recorded every 2 days to generate tumor growth curves. The tumor volumes were determined by measuring their length (l) and width (w) and calculating the volume (V) as the formula: V = lw^2^/2. After tumor growth assessment, the tumors were excised and snap-frozen for RNA and protein extraction or paraffin-embedded for IHC staining.

### In vitro phosphorylation assay

As previously described [27], the phosphorylation reactions were performed in 50 µl kinase buffer (50 mM Tris–HCl PH 8.0, 1% SDS, 1 mM EDTA, 5 mM DTT, 10 mM PMSF, 1 mM NaF, 1 mM Na_3_VO_4_, and protease inhibitor cocktail) at 30 °C for 30 min by including 0.1 mM ATP and different combinations of 20 µg recombinant PERK protein, 50 ng recombinant or immunopurified eIF2α protein, and 1 µg LINP1 in vitro transcript. The final products were analysed by 10% SDS–PAGE.

### Nuclear-cytosolic fractionation

2.5 × 10^7^ cells were harvested by trypsinization, spun down at 500 g for 5 min and washed twice. Cell pellet was resuspended in 5 volumes of CER buffer supplemented with RNase Inhibitor and vortexed for 5 s to completely resuspend the cell pellet with 15 min incubate on ice. The cytoplasmic components (supernatant) and nuclear components (particles) were seperated after centrifugation at 4 °C 1500 g for 5 min. Subsequently, RNA extraction and qPCR was performed as described above.

### Tunicamycin (TM) treatments

Tunicamycin (TM) (5 mg, BBI) treatment was performed in cSCC cells for 12 h after LINP1 knockdown or overexpression.

### Statistical analysis

SPSS 21.0 (IBM SPSS Inc. Chicago, IL, USA) statistical software was used to analyze the data. Statistical tests were performed for independent-samples with an unpaired t-test or one-way ANOVA tests. All statistical tests incorporated two-tailed tests and homogeneity of variance tests, and were considered to reflect significant differences if *P < 0.05, **P < 0.01, or ***P < 0.001. Details of statistical analyses including sample numbers (*n*) are included in the respective figure legends.

## Results

### LINP1 is highly upregulated in cSCC tumors and cells

**Fig. 1:**
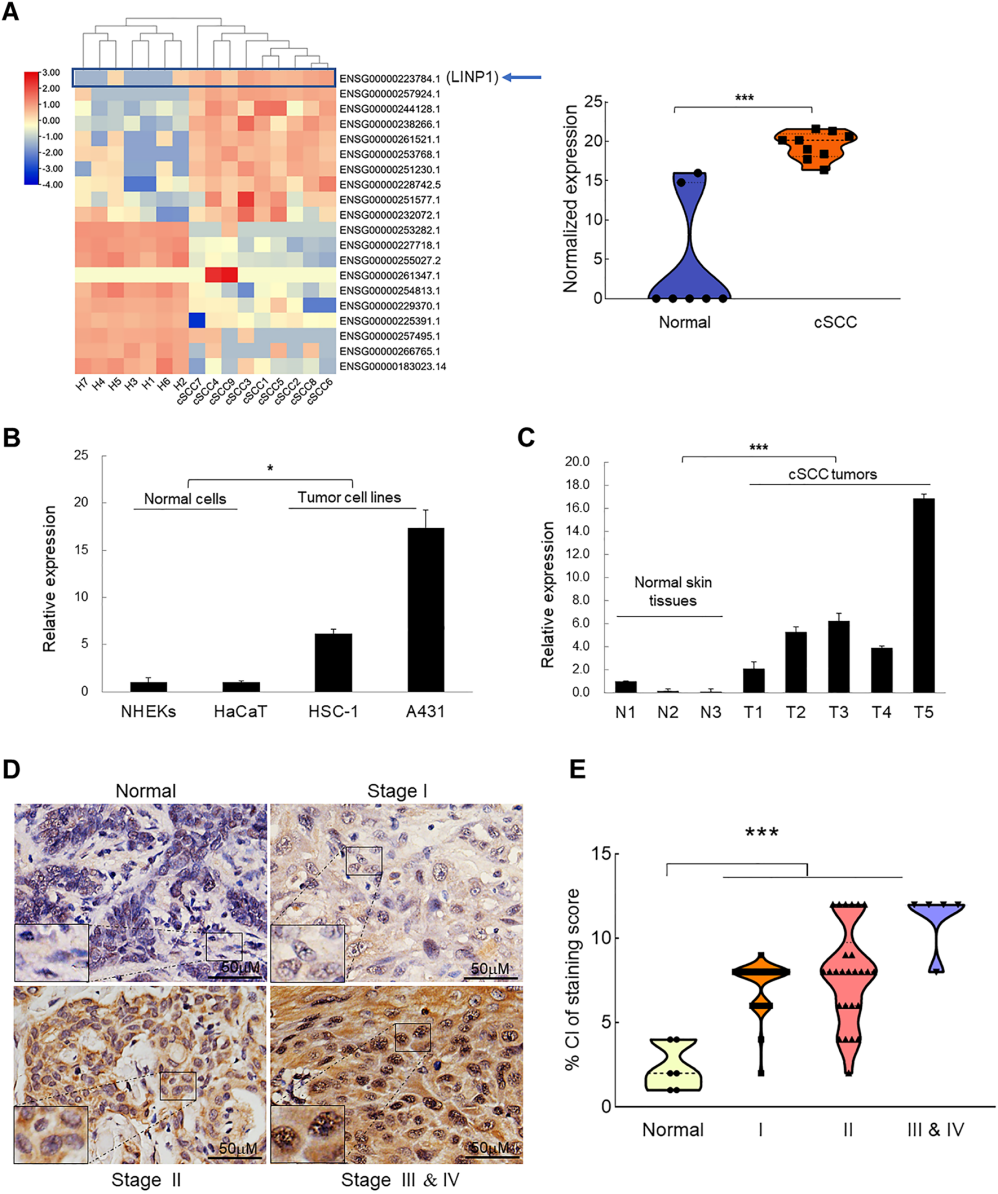
LINP1 is upregulated in cSCC cells and tumors and acts as oncogene. **A** Clustering analysis of top 10 upregulated and top 10 downregulated LncRNAs from published transcriptomic sequencing dataset (GSE139505) [23] was sorted according to the deviation values and shown in a heatmap. Color bars on the left represent ranges of z-value. LINP1 was indicated by a blue arrow. Violin plot was used to compare the normalized expression levels of LINP1 from nine cSCC tissue samples and seven unmatched normal skin samples. **B** The expression levels of LINP1 were detected by qPCR in HaCaT keratinocytes, primary keratinocytes and cSCC cell lines (HSC-1 and A431). **C** The expression levels of LINP1 were compared between normal skin tissues and cSCC tumors. The qPCR data represents the average of three independent experiments ± s.d. **D** ISH detection of LINP1 on paraffin sections of cSCC tumors and normal skin specimens. Representative images with various levels of LINP1 expression (weak staining from normal tissues, stronger straining from tumor tissues) were shown. Scale bar: 50 µm. **E** Association of LINP1 staining scores with tumor grade (Normal skin tissues, I, II, III & IV). *P < 0.05, **P < 0.01, ***P < 0.001

To explore LncRNAs that potentially influence the development of cSCC, we screened one published dataset (GSE139505) based on RNA sequencing of cSCC tumors (n = 9) and healthy skin samples (n = 7) [23]. There are 908 annotated LncRNAs showing significantly altered expression, among which 319 are upregulated and 589 are downregulated in cSCC. Hierarchical Clustering Algorithm after z-score standardization processing of the original normalized expression of the top ten highest upregulated and downregulated LncRNAs in cSCC tumors showed that lncRNA in non-homologous end joining pathway 1 (LINP1) is ranked as the top first LncRNA and significantly higher-expressed in cSCC compared with normal skin tissues (Fig. 1A) [23]. Thus, we chose LINP1 for further analysis.

The higher expression of LINP1 was practically validated by detecting LINP1 in cSCC cell lines (HSC-1 and A431) compared with the primary normal human epidermal keratinocytes (NHEK) and HaCaT keratinocytes (Fig. 1B). To extend this finding to clinical samples, we collected and verified the higher expression of LINP1 in cSCC tumors compared with normal skin tissues (Fig. 1C). To further confirm the higher expression of LINP1 in clinical samples, in situ hybridization (ISH) was performed to detect LINP1 in paraffin-embedded sections of 57 cSCC and 7 normal tissue specimens. Almost all the cSCC samples showed strong LINP1 signal (stronger staining) while lower-level expression of LINP1 could be observed in all normal specimens (Fig. 1D). Further scoring of LINP1 ISH staining sections showed a positive correlation with ascending cSCC grade. Specifically, an evident increasing trend was observed across from normal skin tissue, well-differentiated (Stage I) cSCC (*P* < 0.05), moderately differentiated (Stage II) cSCC to poorly differentiated (Stage III and IV) cSCC (*P* < 0.05) (Fig. 1E). Collectively, the higher expression and tight stage-correlating pattern of LINP1 in cSCC suggests LINP1 might be deeply involved in cSCC progression and potentially play critical functions.

### LINP1 promotes cell proliferation, colony formation, migration and invasiveness in cSCC

**Fig. 2:**
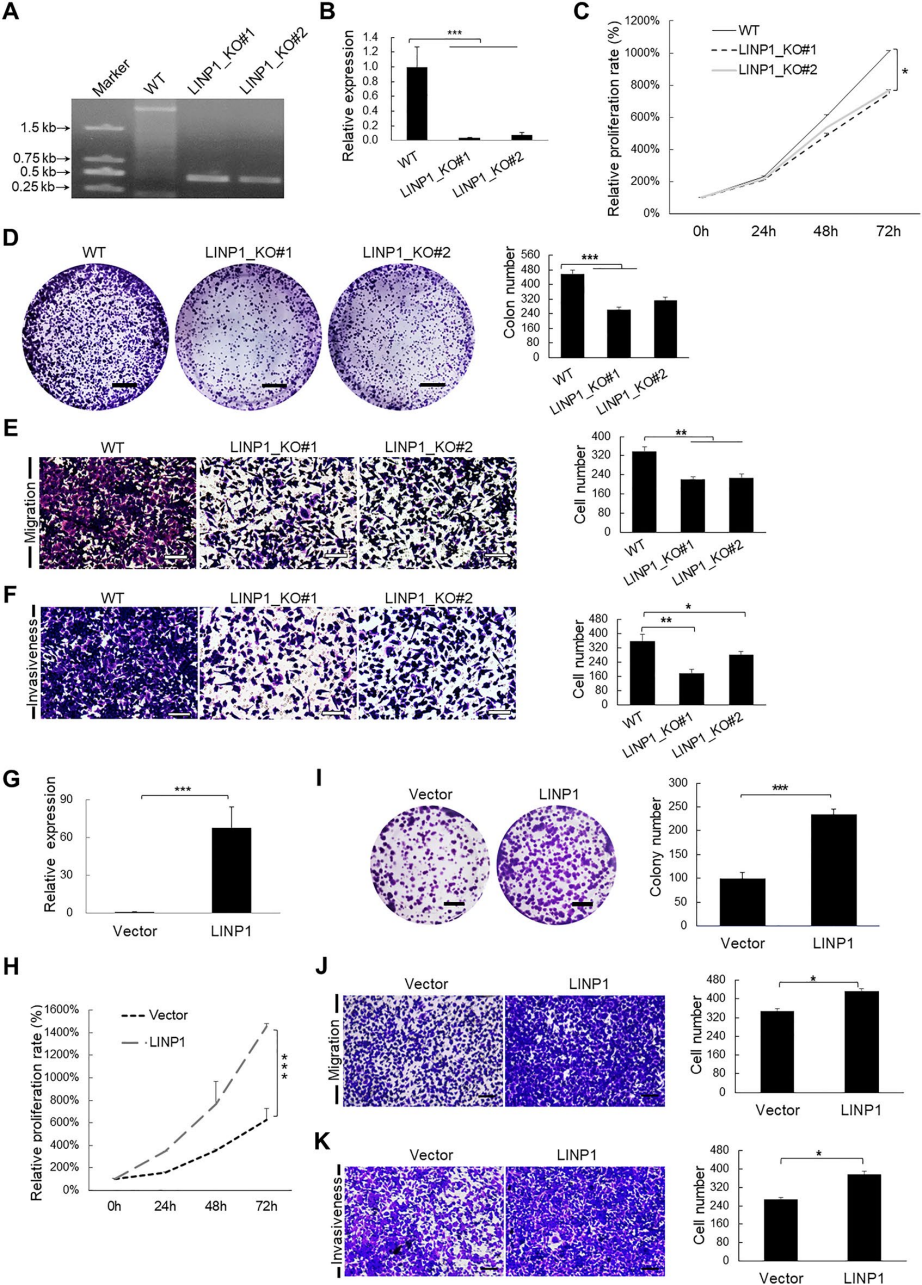
LINP1 promotes cell proliferation, migration and invasiveness in cSCC cells. **A** LINP1 expression was detected after knockout of LINP1 by CRISP/Cas9 technique in HSC-1 cells. **B** Genomic detections in wildtype and knockout cell strains using primer pair located up- and down-stream of LINP1 locus. Measurements of cell proliferation by CCK-8 assay **C**, colony formation assay **D**, transwell migration assay **E** and Matrigel invasiveness measurement **F** were performed in HSC-1 cells treated with siRNAs targeting LINP1. **G** LINP1 expression was detected by qPCR after overexpression of LINP1 in HSC-1 cells. Measurements of cell proliferation by CCK-8 assay **H**, colony formation assay **I**, transwell migration assay **J** and Matrigel invasiveness measurement **K** were performed in HSC-1 cells overexpressing LINP1. Scale bars, 500 mm **C**, **I**, 100 μm **E**, **F**, **J**, **K**. Each experiment was performed in at least triplicate and results are presented as mean ± s.d. One-way ANOVA and Dunnett’s multiple comparison test were used to analyze the data (*P < 0.05, **P < 0.01, ***P < 0.001)

Through searching The Cancer Genome Atlas (TCGA) database, we found LINP1 is highly upregulated in most cancers (80%) relative to corresponding normal tissues (Additional file 1: Fig. S1). Such results with the significant upregulation of LINP1 in cSCC tumors and cells indicate LINP1 might possess an oncogenic role in cSCC development. To test this notion, we prepared LINP1 knockout cSCC cell strains using CRISPR/Cas9 technique and verified the LINP1 knockout efficiency (Fig. 2A, B). LINP1 knockout drastically compromised cell proliferation capacity (Fig. 2C) as shown by CCK-8 assays, which is further supported by colony forming assays indicating fewer colonies formation in LINP1 knockout strains compared with wildtype (WT) strain (Fig. 2D). Transwell migration assays showed that the mobility of cSCC cells was significantly decreased in response to the knockout of LINP1 (Fig. 2E). Invasiveness measurement by Matrigel-coated Transwell indicated that knockout of LINP1 also markedly compromised the invasive capacity of cSCC cells (Fig. 2F). Consistently, the significant-depletion of LINP1 by RNA interference in HSC-1 (Additional file 1: Fig. S2A–E) and in A431 (Additional file 1: Fig. S3A–E) led to drastic compromise of cell proliferation, colony formation, migration and invasiveness. Conversely, overexpression of LINP1 promoted cSCC cell proliferation, colony formation, migration and invasiveness compared with group transfected with empty vector (Fig. 2G–K, Additional file 1: Fig. S3F–J). Collectively, our data demonstrated that LINP1 plays an oncogenic role in promoting cell proliferation, colony formation, migration and invasiveness in cSCC.

### Transcriptomic sequencing reveals LINP1 negatively regulates unfolded protein response and apoptosis signaling

**Fig. 3:**
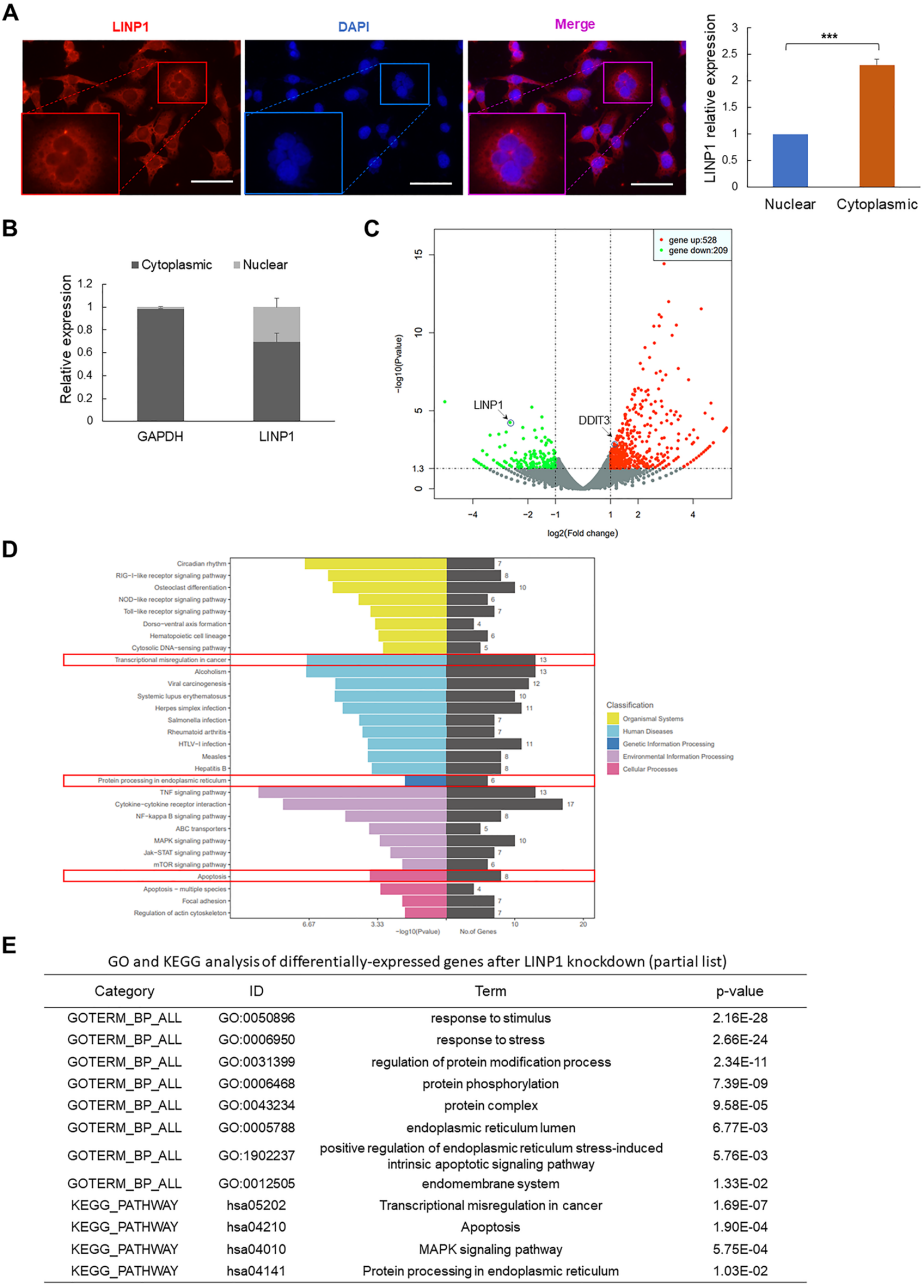
Genome-wide analysis of LINP1-regulated transcriptomic changes by RNA-Seq in cSCC cells. **A** Visualization of LINP1 in HSC-1 cells by RNA fluorescence in situ hybridization (FISH) and quantitative analysis of the ratio of LINP1 in the cytoplasm and nucleus. Scale bars, 50 μm. **B** After isolating the cytoplasmic RNA and nuclear RNA of HSC-1 cells, qRT-PCR was performed to detect the portions of LINP1 in cytoplasm and nucleus. **C** Total RNAs were isolated from HSC-1 cells treated with siNC or siLINP1 oligos and subjected to sequencing. Differentially expressed genes between siNC-treated and siLINP1-treated HSC-1 cells were determined by RNA-Seq and shown by volcano plot. The dots indicating the normalized expression of LINP1 and DDIT3 were shown. **D** Kyoto Encyclopedia of Genes and Genomes (KEGG) pathway analysis of the differentially-expressed genes after LINP1 knockdown. “Protein processing in endoplasmic reticulum”, “Transcriptional misregulation in cancer” and “Apoptosis” were highlighted. Color bars at the right represent gene clusters established through k-means clustering. **E** Gene Ontology (GO) and Kyoto Encyclopedia of Genes and Genomes (KEGG) analysis of the differentially-expressed genes after LINP1 knockdown. Unfolded protein response and apoptosis-related categories are listed

LINP1 was initially identified in triple-negative breast cancer (TNBC) to be functional in non-homologous end joining (NHEJ) by serving as a scaffold to connect Ku80 and DNA-PKCs for enhancing double-strand DNA break repair [24]. However, the subcellular localization of LINP1 reported is mainly in the cytoplasm [24, 25], which is not fully coincident with the nuclear localization required for DNA damage repair [24]. Thus, we also verified the subcellular localization of LINP1 by fluorescent in situ hybridization (FISH) and nuclear-cytoplasmic fractionation. The results indicated LINP1 localizes mainly in the cytoplasm while apparent smaller portion of LINP1 is indeed in the nucleus (Fig. 3A, B), which is quite consistent with the previous reports in other cancers [24, 25].

Since LINP1 is mainly located in the cytoplasm rather than the nucleus, we guess the role of LINP1 in cSCC should be quite different from the role depicted in TNBC [24]. To probe the function executed by LINP1 in cSCC, we performed transcriptomic sequencing (RNA-Seq) to detect the key downstream signaling events after depletion of LINP1 in cSCC cells. Based on the criteria (fold change > 1.5, *P*-value < 0.05), 528 upregulated and 209 downregulated genes were identified in response to LINP1 knockdown (Fig. 3C, Additional file 2: Table S1). The potential signaling pathways were identified by analyzing the significantly-upregulated gene list with the DAVID (The database for annotation, visualization, and integrated discovery, http://david.abcc.ncifcrf.gov/) including KEGG (The Kyoto Encyclopedia of Genes and Genomes) and GO (Gene ontology) modules. The results showed that the lists of enriched GO categories include “protein binding”, “response to stimulus”, “response to stress”, “regulation of protein modification process”, “protein-DNA complex”, “protein complex” and “endoplasmic reticulum lumen”. Strikingly, KEGG pathway analysis indicated that important molecular pathways including “Transcriptional misregulation in cancer”, “Apoptosis” and “Protein processing in endoplasmic reticulum” were significantly enriched and all included a common gene coding transcriptional factor DDIT3 (CHOP), which is critical for UPR-mediated apoptosis (Fig. 3D, E, Additional file 3: Table S2). In summary, LINP1 is mainly localized in cytoplasm and potentially regulates UPR and apoptosis.

### LINP1 functions in repressing UPR-mediated apoptosis

**Fig. 4:**
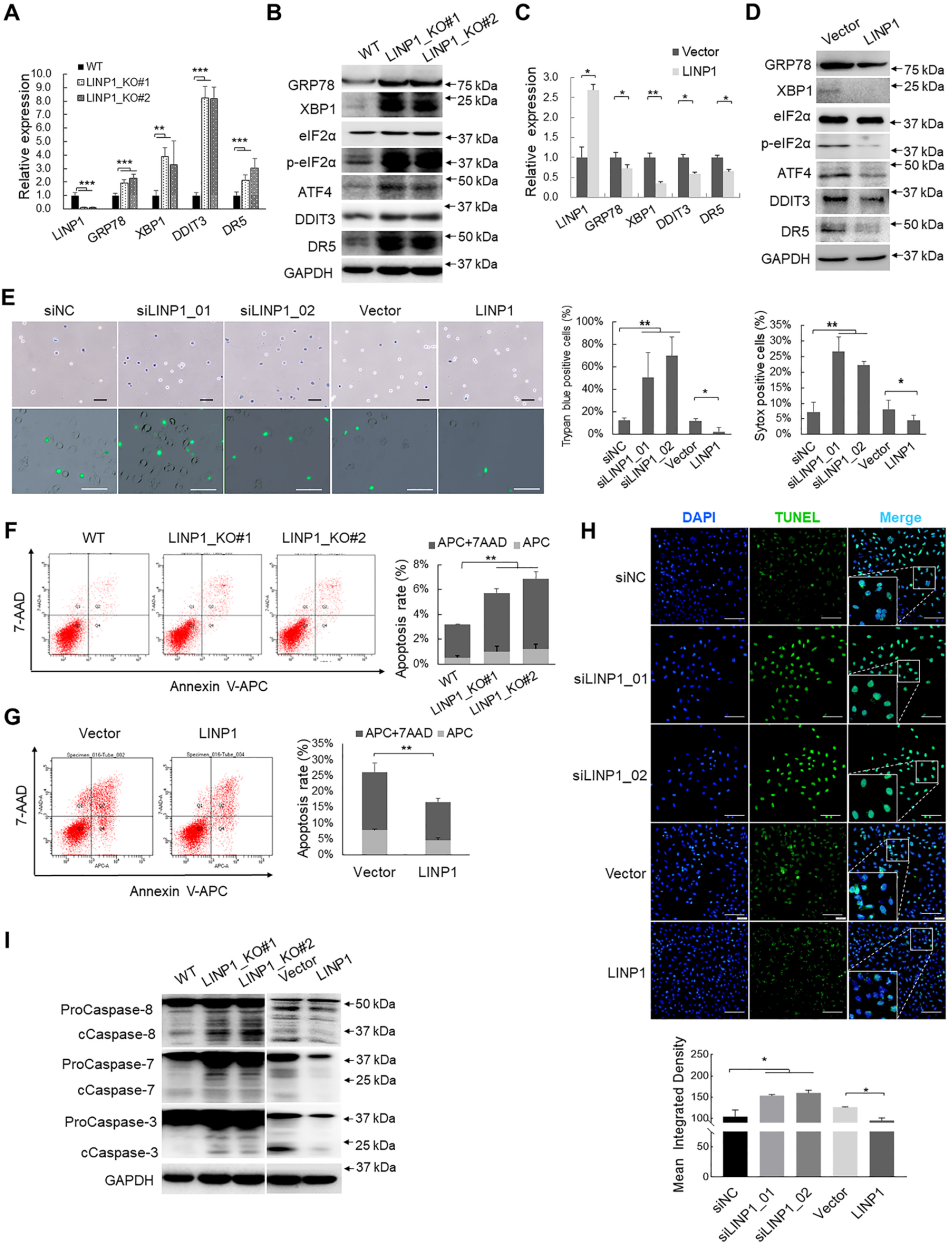
LINP1 functions in repressing UPR and downstream apoptotic genes. **A**, **C** qPCR validations of key gene expression in endoplasmic reticulum signaling including GRP78, XBP1, DDIT3 and DR5 in response to LINP1 knockout and overexpression. Each experiment was performed in at least triplicate and results are presented as mean ± s.d. One-way ANOVA and Dunnett’s multiple comparison test were used to analyze the data (*P < 0.05, **P < 0.01, ***P < 0.001). **B**, **D** Verifications of key protein expression in endoplasmic reticulum signaling including p-eIF2α, GRP78, XBP1, ATF4, DDIT3 and DR5 in response to LINP1 knockout and overexpression by Western blot. **E** Trypan blue staining and Sytox Green staining were performed to evaluate the cell death induced by LINP1 depletion and overexpression in cSCC cells. Scale bars, 50 μm. **F**, **G** Apoptosis assay by Annexin V-APC/7-AAD double staining was performed in cSCC cells after LINP1 knockdown or overexpression. **H** TUNEL assay was performed to detect apoptosis after LINP1 knockdown or overexpression. Scale bars, 100 μm. **I** ProCaspase-8, cleaved Caspase-8, proCaspase-7, cleaved Caspase-7, proCaspase-3 and cleaved Caspase-3 were detected by Western blot in cSCC cells after LINP1 depletion or overexpression. *P < 0.05, **P < 0.01, ***P < 0.001)

DDIT3 is the core transcription factor functioning in driving the transcriptional response of PERK branch of UPR [1]. To verify whether LINP1 modulates UPR signaling, we first checked DDIT3 and typical UPR markers including eIF2α, GRP78, XBP1 and ATF4. Knockout of LINP1 (Fig. 4A, B) and knockdown of LINP1 (Additional file 1: Fig. S2G, H, S4A–B) led to significant upregulation of the mRNA and protein expression levels of GRP78, XBP1 and DDIT3, while LINP1 overexpression repressed their expression (Fig. 4C, D, Additional file 1: Fig. S4A, C). Critically, Knockout of LINP1 (Fig. 4B) and LINP1 depletion (Additional file 1: Fig. S2H, Fig. S4A) enhanced the phosphorylation of eIF2α at Ser 51 site and ATF4 protein expression while overexpression of LINP1 inhibited eIF2α phosphorylation and suppressed ATF4 protein expression (Fig. 4D, Additional file 1: Fig. S4A). As control, the total eIF2α expression levels were not influenced (Fig. 4B, D, Additional file 1: Fig. S2H, Fig. S4A). Thus, consistent with RNA-Seq analysis, LINP1 indeed regulates PERK/eIF2α branch of UPR.

Since the original function of UPR is to rescue cells from ER stress, we sought to discriminate whether LINP1 is pro-survival or pro-apoptosis. Trypan blue exclusion assay and Sytox Green (a nucleic dye excluded by live cells) staining showed that knockdown of LINP1 induced significant increase of cell death rate, while overexpression of LINP1 inhibited the induction of cell death compared with control group (Fig. 4E). Flow cytometry with APC/7-AAD or Annexin V-FITC/PI double staining analysis showed that knockout of LINP1 (Fig. 4F) and knockdown of LINP1 (Additional file 1: Fig. S2F, Fig. S4E) significantly increased the proportion of apoptotic cells, while overexpressing LINP1 inhibited apoptosis (Fig. 4G, Additional file 1: Fig. S4F). Terminal deoxynucleotidyl transferase-mediated biotin-dUTP nick end labeling (TUNEL) assay confirmed the anti-apoptotic role of LINP1 by gain-of- and loss-of-function evaluations (Fig. 4H). Persistent UPR induces apoptosis via activation of death receptor 5 [DR5, also called TNF receptor superfamily member 10b (TNFRSF10B)], which is transcriptionally modulated by UPR mediator DDIT3 and integrates UPR-mediated apoptosis engagement via Caspase-8 [9]. Our results showed that the expression of DR5 was upregulated and the cleaved Caspase-8, Caspase-7 and Caspase-3 were all enhanced in response to LINP1 knockout (Fig. 4A, B, I) and knockdown of LINP1 (Additional file 1: Fig. S2G, H, Fig. S4A, B, D), while overexpression of LINP1 repressed the DR5 expression and the cleavage of Caspase-8, Caspase-7 and Caspase-3 (Fig. 4C, D, I, Additional file 1: Fig. S4A, C, D). Taken together, the above findings indicated that LINP1 inhibits UPR and its downstream apoptosis signaling.

### LINP1 directly interacts with eIF2α to protect eIF2α from phosphorylation at Ser51 by PERK

**Fig. 5:**
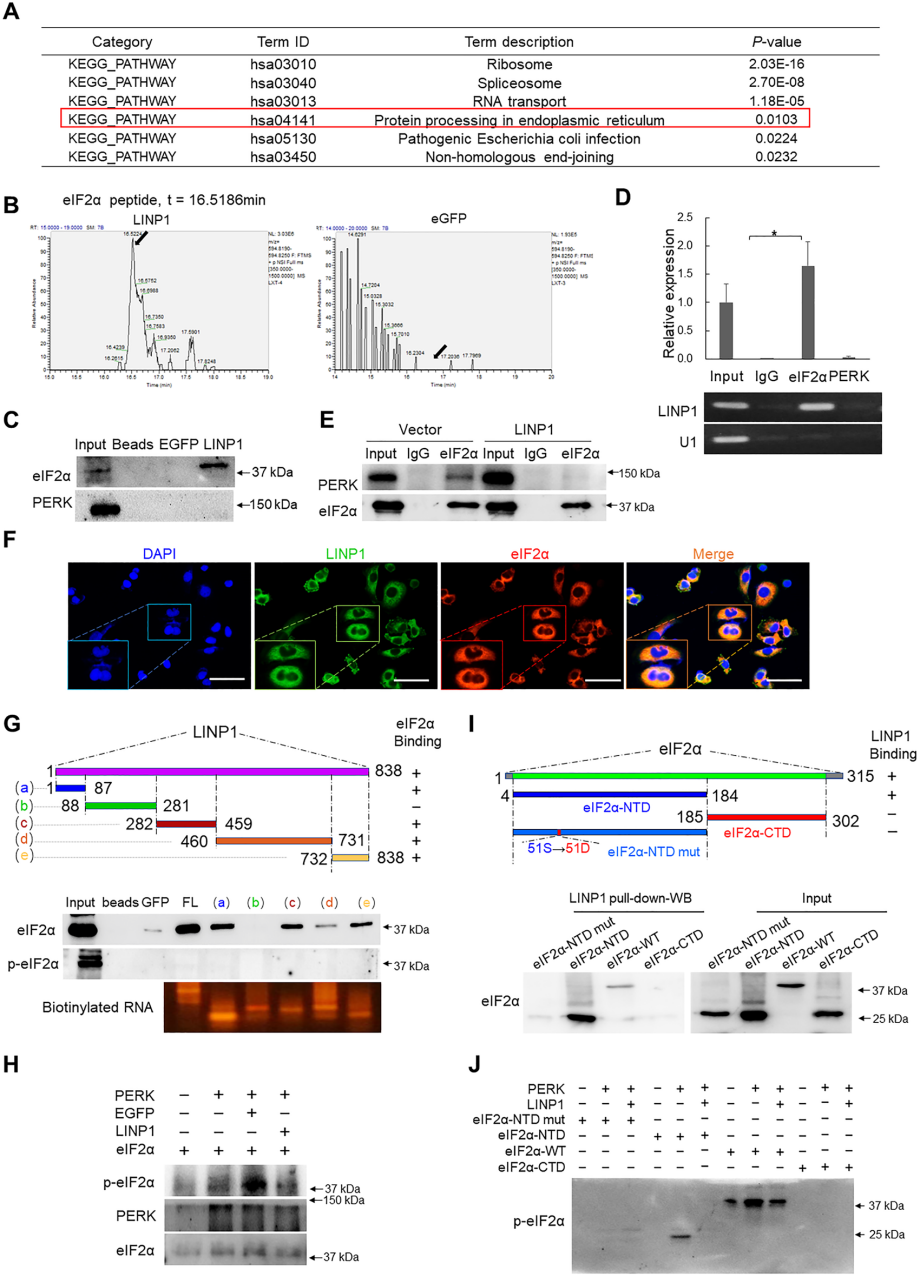
LINP1 directly interacts with eIF2α to protect eIF2α from phosphorylation at Ser51 by PERK. **A** Kyoto Encyclopedia of Genes and Genomes (KEGG) pathway analysis of the proteins interacting with LINP1 by subtracting the proteins non-specific binding to EGFP RNA after identified by HPLC–MS. “Protein processing in endoplasmic reticulum” is highlighted. **B** Arrows indicate the identified eIF2α peptide peak in the LINP1-pulldown sample, which was lacking in control eGFP sample. **C** Biotin-labeled LINP1 transcript was used to retrieve interacting protein partners by RNA pulldown with beads only and EGFP RNA as controls. The resulting protein mix from cSCC cells was applied to detect eIF2α and PERK by Western blot. **D** RNA immunoprecipitation (RIP) assay was performed using antibodies against eIF2α and PERK while IgG was used as control. The retrieved LINP1 RNA was detected by qPCR. *U1* transcripts were used as a negative control. **E** Co-IP of PERK and eIF2α  followed by Western blot was performed to check whether overexpression of LINP1 influences the interaction between PERK with eIF2α. **F** Fluorescence in situ hybridization (FISH) and immunofluorescence (IF) were performed to examine the co-localization of LINP1 (green) and eIF2α (red) in cSCC cells. Scale bars, 50 μm. Quantitative analysis of the fluorescence co-localization of eIF2α and LINP1 was performed. Pearson’s *R* value = 0.89. **G** Biotin-labeled full-length (FL) or domain fragments of LINP1 transcripts were used to retrieve eIF2α or PERK proteins and Western blot was performed. **H** In vitro phosphorylation reactions containing recombinant bacterially expressed GST–PERK was done in 50 µl kinase buffer with 0.1 mM ATP and 50 ng partially purified eIF2α. The impact of LINP1 on eIF2α phosphorylation by PERK was evaluated by adding 1 µg LINP1 transcript to the reactions. The products were analyzed by 10% SDS–PAGE. **I** In vitro pulldown of in vitro-expressed full-length protein, wild type NTD, mutated NTD domain containing Ser51 to Asp51 mutation and wild type CTD of eIF2α by biotinylated LINP1 transcripts were examined by Western blotting. **J** In vitro phosphorylation assay containing GST–PERK (20 µg), 0.1 mM ATP and 1 µg LINP1 transcript were done in 50 µl kinase buffer with 50 ng Flag-tagged in vitro-expressed full-length protein, wild type of NTD, mutated NTD domain containing Ser51 to Asp51 mutation and wild type of CTD

Since LINP1 is mainly localized in cytoplasm, the functional mechanism of LINP1 in cSCC should be quite different from the role in TNBC. As LncRNAs generally associate with proteins to perform their functions [15], we conducted RNA pulldown with in vitro-transcribed and biotin-labeled full-length LINP1 RNA and control EGFP RNA to probe the potential interacting protein partners of LINP1. The specific binding protein profile of LINP1 was identified by high-performance liquid chromatography-mass spectrometry (HPLC-MS) excluding the non-specific binding proteins associated with EGFP RNA (Additional file 4: Table S3). Such list was analyzed by Kyoto Encyclopedia of Genes and Genomes (KEGG) and the clustered KEGG categories include “Ribosome”, “Spliceosome”, “RNA transport”, “Protein processing in endoplasmic reticulum”, “Pathogenic *Escherichia coli* infection” and “Non-homologous end-joining” (Fig. 5A, Additional file 5: Table S4). Two of the characterized proteins, XRCC5 (KU80) and PRKDC (DNA-PKcs), belong to the category “Non-homologous end-joining”, which is consistent with previous reports [24] and demonstrates the reliability of our assays. Interestingly, the category “Protein processing in endoplasmic reticulum” is quite in line with the role of UPR-suppressive LINP1 identified in Fig. 4. More importantly, one protein among this category is eukaryotic translation initiation factor EIF2S1 (eIF2α) (Fig. 5B, Additional file 1: Fig. S5A), which is critical for the initiation of PERK branch of UPR. Consistent with the results of HPLC-MS analysis, the direct association probability of LINP1 and eIF2α we predicted through the LncPRO (http://bioinfo.bjmu.edu.cn/lncpro/) [26] is 88.539 (The score is between 0 and 100 and the threshold is 50) (Additional file 1: Fig. S5B). The predicted interaction score between LINP1 and eIF2α is far above 50, suggesting eIF2α is very likely to associate with LINP1.

To verify the relationship between LINP1 and eIF2α, RNA pulldown using biotinylated RNA and RNA immunoprecipitation using specific antibodies were performed and confirmed LINP1 physically interacts with eIF2α (Fig. 5C, D). Since eIF2α is the casual substrate supposed to be phosphorylated by PERK [9], the potential interaction between LINP1 and PERK was also verified. However, LINP1 did not show any interaction with PERK (Fig. 5C, D). To detect if LINP1 regulates eIF2α interaction with PERK, we overexpressed LINP1 and performed eIF2α immunoprecipitation and the results showed that LINP1 overexpression interfered the interaction between eIF2α and PERK compared with vector control (Fig. 5E). The interaction between LINP1 and eIF2α was also supported by co-localization detection by confocal microscopy using fluorescence in situ hybridization (FISH) for LINP1 and immunofluorescence (IF) staining for eIF2α respectively. Most of the eIF2α signal (Red) was co-localized with LINP1 signal (Green) and quantitative analysis of the co-localization showed that the Pearson’s correlation coefficient was 0.89 (Fig. 5F). Thus, the above analysis strongly demonstrated the direct association between LINP1 and eIF2α.

To further map the domain of LINP1 directly binding with eIF2α, we analyzed the predicted secondary structure of LINP1 by RNAfold (Additional file 1: Fig. S5C) and generated a series of deletion mutants of LINP1 (Fig. 5G). Full-length LINP1 in RNA pulldown experiment showed the strongest bind intensity and different fragments possess differential binding capacities as indicated by the strong binding of fragments 1, 3 and 5 and weak binding of fragment 4 while fragment 2 retrieved no eIF2α (Fig. 5G). The above results indicated the interaction between LINP1 and eIF2α maybe require the participation of separate domains of LINP1.

Such unique binding features with the phosphorylation-suppressive function of LINP1 to eIF2α (Fig. 4B, D) led us curious to guess that LINP1 may directly modulate the phosphorylation of eIF2α. To validate such a hypothesis, we performed in vitro phosphorylation experiment using bioengineering-expressed full-length PERK protein and immunoprecipitated eIF2α protein from cSCC cells together with in vitro-transcribed LINP1 in kinase buffer system for in vitro phosphorylation assay of eIF2α [27]. Originally, the immunoprecipitated cellular eIF2α exhibited basic phosphorylation which is apparently enhanced in the presence of PERK (Fig. 5H). Further, LINP1 significantly inhibited eIF2α phosphorylation compared with control EGFP RNA (Fig. 5H). To explore the binding domain of eIF2α with LINP1 and validate whether LINP1 directly regulates eIF2α phosphorylation, full-length eIF2α was separated to N-terminal domain (NTD, residues 4–184) containing the unique Ser51 phosphorylation site and C-terminal domain (CTD, residues 185–302) according to previous reports [10]. The in vitro RNA pulldown was performed using in vitro-expressed full-length eIF2α protein, wild type of NTD, mutated NTD domain containing Ser51 to Asp51 mutation and wild type of CTD. The results showed that full-length eIF2α and N-terminal domain (NTD, residues 4–184) containing the unique Ser51 phosphorylation site strongly interact with LINP1 while mutated NTD domain and wild type of CTD did not (Fig. 5I). Next, we performed in vitro phosphorylation using the above in vitro-expressed full-length and different domains of eIF2α together with PERK to investigate the influence of LINP1 on eIF2α phosphorylation. PERK strongly promoted the phosphorylation of full-length eIF2α protein while LINP1 significantly suppressed eIF2α phosphorylation (Fig. 5J). Similar performance of LINP1 in inhibiting Ser51 phosphorylation was repeated and looked even more obvious using wild type of NTD (Fig. 5J) while almost no phosphorylation could be detected using mutated NTD and wild type of CTD (Fig. 5J). Collectively, LINP1 directly interacts with the NTD of eIF2α at Ser51 to repress the eIF2α phosphorylation.

### The UPR-induced apoptosis signaling modulated by LINP1 is dependent on DDIT3

**Fig. 6:**
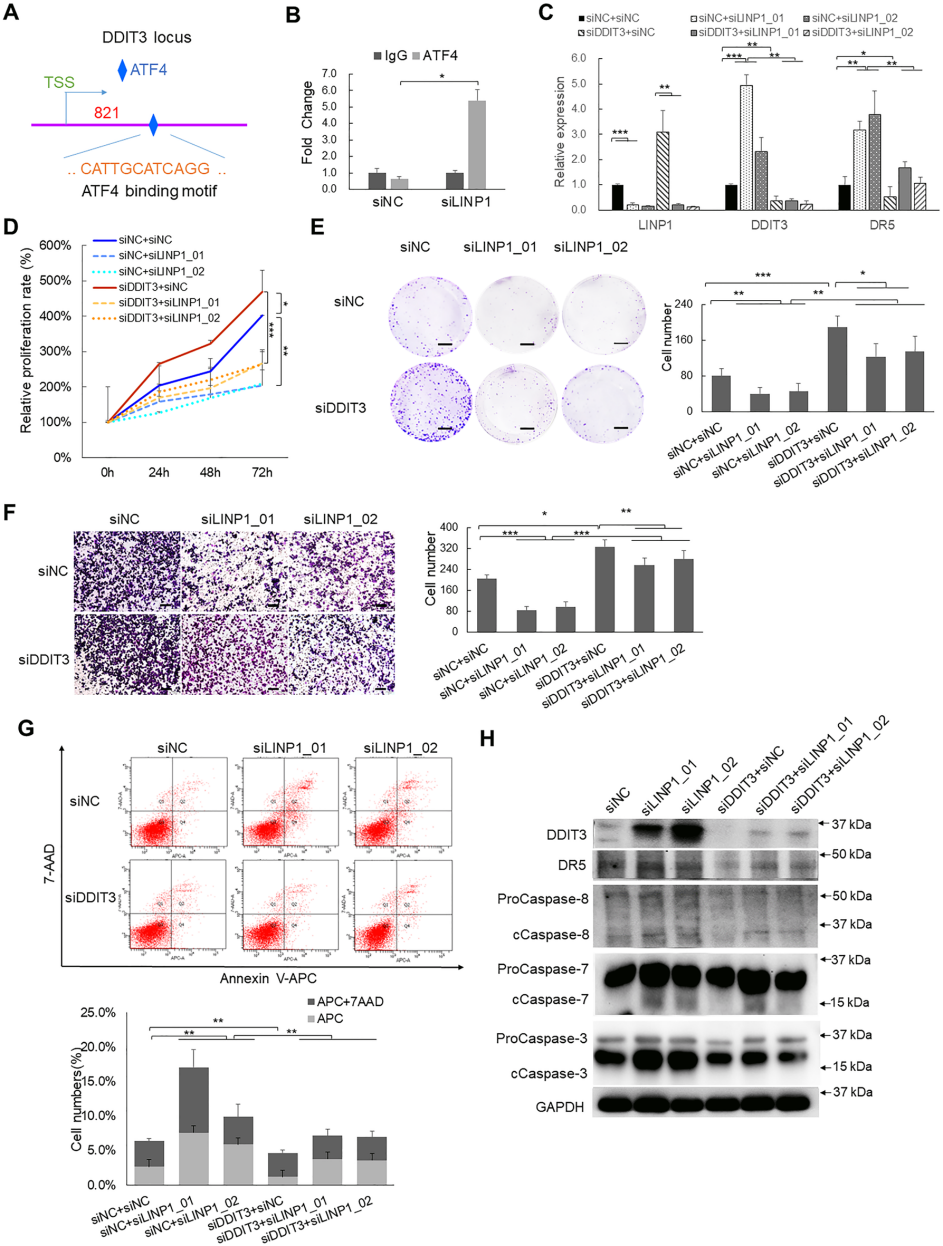
The UPR-mediated apoptosis modulated by LINP1 is dependent on DDIT3. **A** The binding site of ATF4 on the promoter region of DDIT3 was predicted by rVista (https://rvista.dcode.org/) and UCSC genome browser (https://genome.ucsc.edu/). **B** The binding enrichment of ATF4 at the binding site on the promoter region of DDIT3 was detected by ChIP-qPCR after depletion of LINP1. **C** LINP1, DDIT3 and DR5 RNA expression were detected by qPCR after LINP1 knockdown and/or DDIT3 knockdown. **D**–**G** Cell proliferation, colony formation assay, migration, invasiveness assays and Annexin V-APC/7-AAD double staining measurement were performed after depletion of LINP1 or/and DDIT3. **H** The protein levels of DDIT3, DR5 and cleaved forms of Caspase-8, Caspase-7 and Caspase-3 were detected. Each experiment was performed in at least triplicate and results are presented as mean ± s.d. One-way ANOVA and Dunnett’s multiple comparison test were used to analyze the data. (*P < 0.05, **P < 0.01, ***P < 0.001). Scale bars, 500 mm **E**, 100 μm **F**

As the key step of UPR, phosphorylation of eIF2α induces the translation of ATF4 which is then translocated to and enriched at the promoter of DDIT3 gene locus to induce DDIT3 expression for activating downstream gene expression [28, 29]. To investigate whether the translocation of ATF4 was indeed take placed after LINP1 depletion, we performed chromatin immunoprecipitation (ChIP) followed by qPCR and the results clearly showed that the binding enrichment of ATF4 was significantly enhanced in response to loss of LINP1 (Fig. 6A, B).

As we already confirmed the negative regulation of key transcription factor DDIT3 by LINP1 (Fig. 4A–D), we would like to ask whether the oncogenic and anti-apoptotic functions of LINP1 are both dependent on the suppression of DDIT3. To examine this hypothesis, we first verified whether DDIT3 regulates UPR-induced apoptosis. We depleted DDIT3 by RNA interference using siRNAs targeting DDIT3 in cSCC cells (Additional file 1: Fig. S6A). Knockdown of DDIT3 significantly promoted cell proliferation, colony formation and migration (Additional file 1: Fig. S6B–D) but compromised apoptosis (Additional file 1: Fig. S6E). We also observed that knockdown of DDIT3 repressed the DR5 expression and the cleavage of Caspase-8, Caspase-7 and Caspase-3 (Additional file 1: Fig. S6F). The above results demonstrated the pro-apoptotic function of DDIT3 in cSCC.

Further analysis by double depletions of both LINP1 and DDIT3 showed that knockdown of DDIT3 could partially rescue LINP1 depletion-induced apoptosis and partially restore the cell viability, colony formation, migration back to the original levels (Fig. 6C–H), which further demonstrated the tight upstream and downstream relationship between LINP1 and DDIT3. Thus, the above results suggested that LINP1 performs oncogenic and apoptosis-suppressive roles through DDIT3.

### LINP1 suppresses PERK/eIF2α axis-modulated UPR signaling and the following apoptosis

**Fig. 7:**
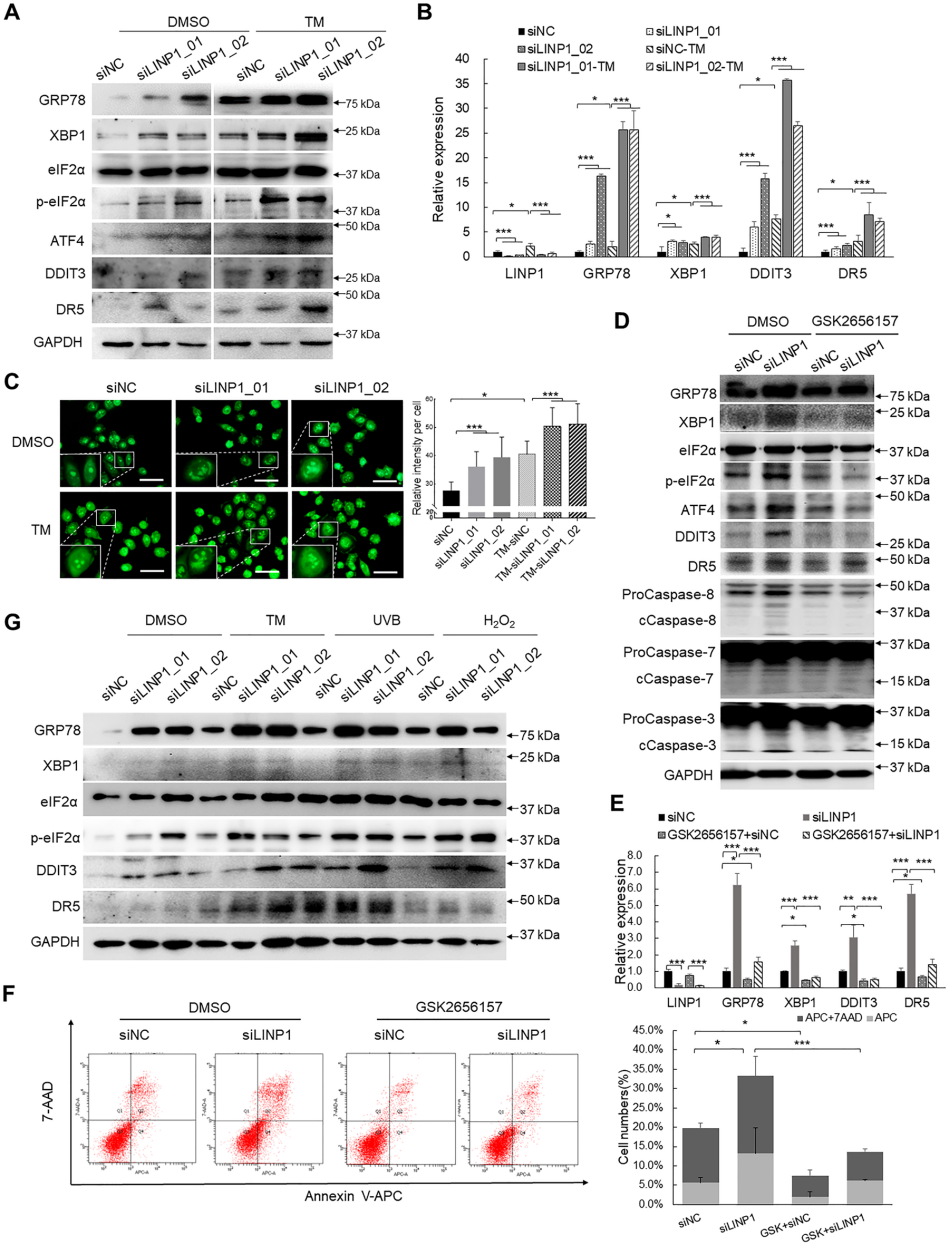
LINP1 suppresses PERK/eIF2α axis-modulated UPR signaling and the following apoptosis.. **A** Detection of key protein expression in UPR signaling including p-eIF2α, GRP78, XBP1, ATF4, DDIT3 and DR5 in response to LINP1 knockdown under TM (2 µg/mL) treatment by Western blot. **B** Detection of key gene expression in UPR signaling including GRP78, XBP1, DDIT3 and DR5 in response to LINP1 knockdown under TM (2 µg/mL) treatment by qPCR. **C** Detection of ThT fluorescence intensity corresponding to ER stress-induced activation of the unfolded protein response after TM treatment and LINP1 knockdown. Scale bars, 50 μm. **D**, **E** After LINP1 depletion, cSCC cells was pretreated with PERK inhibitor GSK2656157 for 1 h and then treated with 2 µg/mL TM for 12 h. The expression level of the UPR signaling genes were determined by Western blot and qPCR. **F** Apoptosis rate was detected by Annexin V-APC/7-AAD double staining after LINP1 knockdown. Data are plotted as the means ± s.d. n = 3. *P < 0.05, **P < 0.01, ***P < 0.001. **G** Detection of key protein expression in UPR signaling including p-eIF2α, GRP78, XBP1, ATF4, DDIT3 and DR5 in response to LINP1 knockdown in HaCaT cells under the treatments of TM, ultraviolet B and H_2_O_2_

Since UPR is generally induced by ER stress, one reasonable question would be asked, whether LINP1 functions in common ER stress-induced response? To address this concern, we established known ER stress models using known ER stress inducer tunicamycin (TM). TM treatment did enhance eIF2α phosphorylation and upregulated expression of DDIT3, XBP1, GRP78, ATF4 and DR5 in cSCC cells, while depletion of LINP1 further promoted eIF2α phosphorylation and upregulation of the above PERK branch factors in both mRNA and protein levels compared with controls (Fig. 7A, B). Notably, the expression of total eIF2α was not significantly changed (Fig. 7A).

Thioflavin T (ThT) and its derivatives are highly affinitive to misfolded protein and act as good indicators of ER stress to quantify ER stress-induced protein aggregation within cells [30–32]. We subsequently tested the role of LINP1 in TM-induced protein aggregation using ThT. In addition to the marked enhancement of ThT fluorescence intensity indicating protein aggregation after TM treatment, LINP1 depletion further strengthened protein aggregation, suggesting that LINP1 depletion can promote ER stress-induced protein aggregation and UPR activation (Fig. 7C).

Regarding PERK is not the only kinase to phosphorylate eIF2α [33], PERK specific inhibitor GSK2656157 was applied to verify the role of LINP1 in PERK/eIF2α signaling axis. CSCC Cells after LINP1 knockdown were pretreated with GSK2656157 (2 μmol/L) for 1 h and followed by exposing to 2 μg/mL TM for 12 h. Compared to control DMSO treatment, inhibition of PERK by GSK2656157 treatment significantly decreased TM-induced eIF2α phosphorylation and upregulation of GRP78, XBP1, ATF4, DDIT3 and DR5 (Fig. 7D, E). In addition to above effects, GSK2656157 treatment repressed LINP1 depletion-induced eIF2α phosphorylation and upregulation of GRP78, XBP1, ATF4, DDIT3 and DR5 (Fig. 7D, E). Consistent with this result, pretreatment with PERK inhibitor obviously attenuated UPR-induced apoptosis and inhibited apoptosis induced by knockdown of LINP1 (Fig. 7D, F). In summary, LINP1 is critical for inhibiting PERK/eIF2α axis-modulated UPR.

To further test the broad applicability of the above findings, we verified the role of LINP1 in keratinocyte cell line HaCaT treated with several kinds of ER stress stimuli including TM, ultraviolet B and H_2_O_2_. Consistently, the phosphorylation of eIF2α and the expression of DDIT3, XBP1, GRP78 and DR5 significantly elevated upon treatments with all three stimuli while loss of LINP1 further reinforced such enhancement (Fig. 7G). In summary, LINP1 definitely inhibits ER stress-induced UPR signaling activated by different kinds of stimuli in keratinocyte lineage.

### *Loss of LINP1 inhibits tumor growth and promotes UPR-induced apoptosis signaling *in vivo

**Fig. 8:**
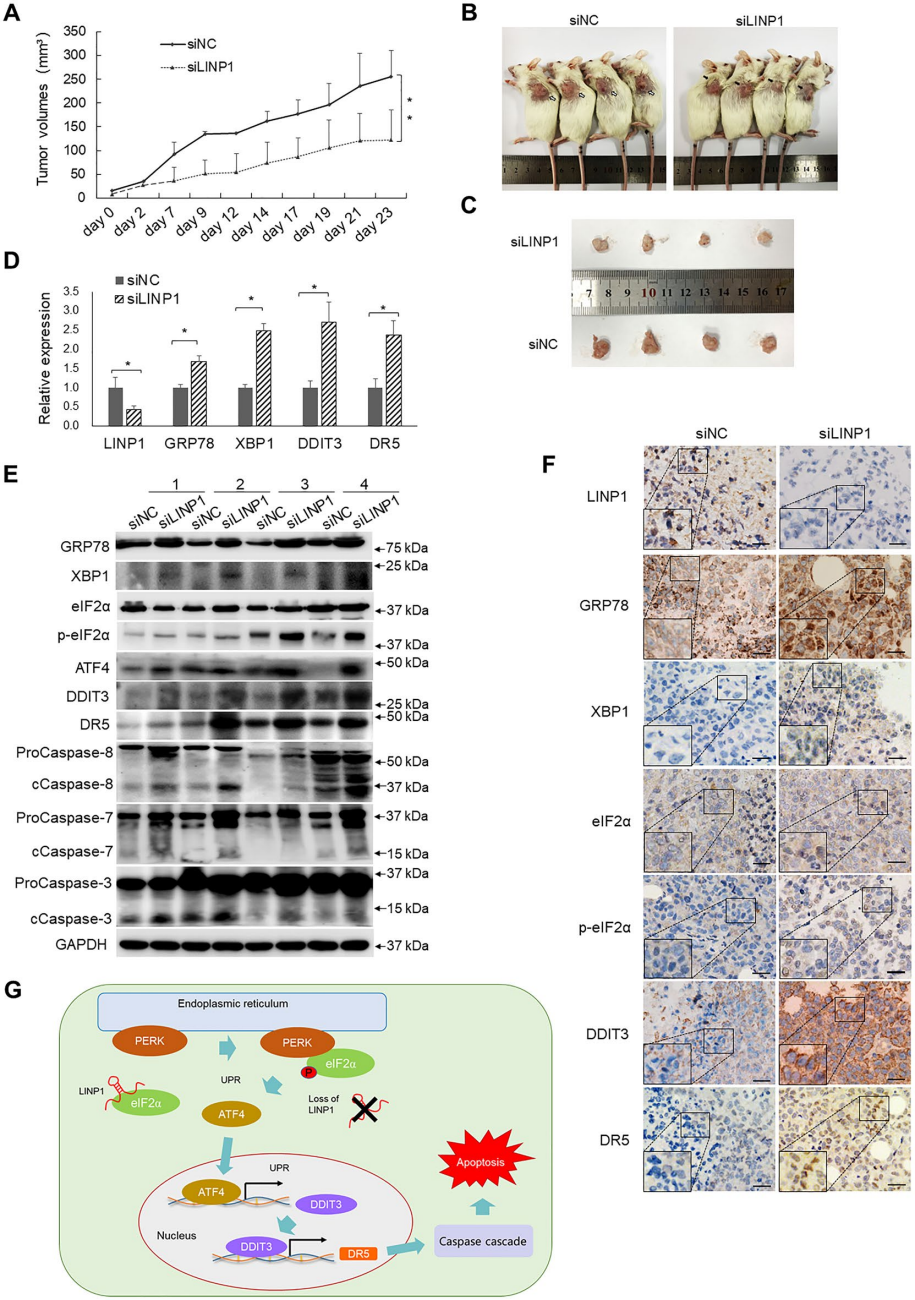
Loss of LINP1 inhibits tumor growth and promotes UPR-mediated apoptosis in vivo. **A** Loss of LINP1 inhibits cSCC growth in a mouse xenograft model. Tumor volumes (mm^3^) were plotted according to day. **B**, **C** The mice were sacrificed at the end of the experiment and the dissected tumors from four mice are shown. White and black arrows respectively indicate the siNC-treated and siLINP1-treated xenografts. **D** The expression of LINP1, GRP78, XBP1, DDIT3 and DR5 were detected in the dissected xenografts by qPCR. Statistical data of qRT-PCR represented the average of four independent experiments ± s.d. **E** The protein levels of eIF2α, p-eIF2α, GRP78, XBP1, DDIT3 and DR5 and cleaved Caspase-8, cleaved Caspase-7, cleave Caspase-3 were detected in xenografts after siLINP1 treatment by Western blot. **F** The expression levels of LINP1, DDIT3, DR5, XBP1 and GRP78 in tumor sections were evaluated using in situ hybridization or IHC staining. Scale bar, 50 µm. **G** A model depicts that LINP1 modulates eIF2α phosphorylation to repress UPR-mediated apoptosis and promote cSCC development

To evaluate the pro-carcinogenic and UPR-suppressive functions of LINP1 in vivo, a xenograft tumor model was established in immunocompromised mice (NCG strain). No significant difference in tumor size could be observed initially between the siNC control group and siLINP1-treated group. After 7 days, loss of LINP1 led to marked loss of size of xenografts (Fig. 8A) and evident smaller tumor mass formation at the end of the evaluation compared with control group (Fig. 8B, C). The knockdown efficiency of LINP1 expression in siLINP1-treated group was verified by qPCR and ISH staining in xenograft tumors (Fig. 8D, F). Importantly, LINP1 depletion led to the significant enhancement of the phosphorylation of eIF2α (Fig. 8E, F), the upregulation of UPR factors GRP78, XBP1, ATF4, DDIT3 and apoptotic factor DR5 (Fig. 8D–F) and the cleavage of caspases including Caspase-8, Caspase-7 and Caspase-3 (Fig. 8E). Collectively, the in vivo experiments indicated that LINP1 suppresses UPR signaling and the following apoptosis by modulating the phosphorylation of eIF2α to promote cSCC progression.

## Discussion

Tumor cells commonly grow in harsh microenvironment and face the challenges of cellular stresses including oxidative stress, metabolic stress, genotoxic stress and ER stress, etc. Properly handling such adverse situations would be beneficial to tumor cells to maintain homeostasis and enhance their survival and further progression [34–36]. However, chronic exposure to stresses or unmitigated responses would compromise the beneficial effects, promote diseases and cancer and even cell death. Tumor cells have to evolve the adaptive capacities to deal with adverse environmental conditions to ensure positive-selection of the survival of stress-adapted cells and negative-selection of damaged cells [37]. Exploring the underlying signaling mechanisms is fascinating to disclose the survival secrets and contribute to develop novel anti-tumor drugs.

Long noncoding RNAs (LncRNAs) are tissue-specifically expressed and play key regulatory roles in important physiological and pathological processes such as carcinogenesis, angiogenesis, muscle development or immune regulation [15, 38]. LncRNAs can act as a scaffold or guide to regulate protein–protein and the following signaling events, as decoys to lower in vivo active concentration of miRNAs, and as chromatin modifiers to modulate gene expression by enhancers [15, 38]. Intensive studies in cancer field have revealed many LncRNAs are widely expressed in tumors and act as oncogene or tumor suppressor to influence tumor progression. Regarding the number of LncRNAs in tens of thousands, their functional mechanisms are far from being fully understood.

LINP1 locates on the short arm of human chromosome 10 with 2 exons. There is almost no homolog of LINP1 in other species except for a predicted non-coding transcript with a homologous region of only 186 bp in length and 84% similarity in cynomolgus monkeys, suggesting that LINP1 and its homolog transcripts may be unique to primates. The lack of homolog in rodents limits the means to validate LINP1 function and mechanism using knockout animal model. LINP1 was first found to be highly expressed in triple-negative breast cancer (TNBC) and participates in non-homologous end joining (NHEJ) pathway by acting as a scaffold to bind the ATP-dependent DNA helicase complex Ku80-Ku70 heterodimer and catalytic subunit DNA-PKcs to the damage site to promote DNA junction repair. Knockdown of LINP1 enhances the sensitivity of TNBC to chemotherapy. In cervical cancer, LINP1 translocates from the cytoplasm to the nucleus to bind Ku80 and DNA-PKcs to promote DNA damage repair during radiotherapy. Loss of LINP1 significantly enhances ionizing radiation-induced apoptosis. In addition, LINP1 is also up-regulated in a variety of cancers, significantly affecting cancer-related processes such as cell proliferation, migration, invasion, and apoptosis and participating in disease pathogenesis. For example, LINP1 is highly expressed in patients with acute myeloid leukemia (AML), but down-regulated in patients with complete remission after treatment; LINP1 enhances HNF4a-AMPK/WNT5A signaling pathway activation and promotes AML progression, and knockdown of LINP1 significantly inhibits AML cells of glucose absorption and survival. LINP1 is highly expressed in esophageal squamous cell carcinoma tissues and cell lines. In vivo and in vitro experiments show that knockdown of LINP1 causes tumor cells to arrest in the G2/M phase, inhibits cell proliferation and in vivo xenograft tumor growth and significantly promotes cell apoptosis. The current in-depth mechanism study of LINP1 is limited to the repair of DNA damage in the NHEJ pathway induced by treatments of ionizing radiation and chemical drugs.

However, the fluorescence in situ hybridization data from two of the above reports clearly indicated LINP1 was mainly located in the cytoplasm of cells and only a small portion in the nucleus. Even in the case of partial LINP1 translocating from the cytoplasm to the nucleus under radiotherapy treatment, the main subcellular localization of LINP1 is still dominated by the cytoplasm. A number of studies have shown that LINP1 can participate in multiple important cellular processes (such as proliferation, migration, invasion, apoptosis, etc.) and play strong regulatory roles without the presence of DNA damage-inducing agents. Such phenomenon suggests that DNA damage repair may be only one of the functions performed by LINP1 in tumor cells and its critical functions in cells are far from full elucidation.

In this study, LINP1 is identified to be significantly higher-expressed in cSCC tumors compared with normal skin tissues and positively correlated with tumor staging. In addition to the confirmation of its oncogenic role in cSCC by routine oncogene verifications, our transcriptomic analysis after LINP1 depletion indicated its function may link with ER stress-induced UPR (Fig. 3D, E). KEGG clustering of proteins identified by RNA pulldown followed by HPLC-MS also indicated UPR signaling-related categories (Fig. 5A, B). Especially, the subcellular localization of LINP1 is mainly in cytoplasm with only a small portion of LINP1 present in nucleus (Fig. 3A, B). These evidences prompted us LINP1 may be tightly involved in ER stress response. Critically, LINP1 is physically associated with the initiator of UPR signaling, eIF2α, which is validated by RNA pulldown and RIP assays (Fig. 5C, D) and spatially supported by confocal microscopy observation (Fig. 5F). Interestingly, further in vitro and in vivo investigations discovered LINP1 not only interacts with eIF2α but also constrains PERK-mediated eIF2α phosphorylation to avoid overaction of UPR and the following apoptosis (Fig. 5H, J). Although previous reports also observed the cytoplasm-dominated subcellular localization pattern of LINP1, they focused on the role of nucleus-localized LINP1 and elucidated that LINP1 associates with Ku80 and DNA-PKcs and functions in NHEJ DNA repair pathway [24, 25]. Our findings replenish the functions of LINP1 in maintaining cell homeostasis and contribute to the full understanding of the diverse roles of LncRNAs.

The regulation of eIF2α phosphorylation depends on the gambling between pro-phosphorylation process by kinases and dephosphorylation by phosphatases [10]. miRNAs and cofactors are indirectly involved in such gambling by targeting kinases or phosphatases [11, 12]. Here, we present a new finding that LINP1 directly attenuates the kinase-mediated phosphorylation of eIF2α and enforces the survival capacity by arming the cell with an extra shield to avoid unmitigated UPR. Such a novel regulatory hierarchy disclosed by this study enriches our understanding about the fine-tuning network in maintaining cellular homeostasis to improve the adaptation ability and survival of cells against harsh microenvironments especially for tumor cells, which may suggest new targets for drug development. This study also reminds us the unlimited potential for LncRNAs and reevaluates their importance in signal transduction and stress responses. Accompanied by the in-depth explorations of LncRNAs, it looks the current theory is not capable to fully predict the exact roles of LncRNAs in different tissues and environment. Post-translational protein modifications influence the structure, electrophilicity and even interaction network of proteins and are responsible for the functional divergence of the same protein. Specially, recent advances  have primarily disclosed the involvement of LncRNAs in modulating protein modifications. PURPL physically interacts with ULK1 and differentially regulates its phosphorylation by promoting the association with mTOR and departure from AMPK to suppress autophagic cell death for maintaining the survivability of melanoma cells [39]. LincRNAFEZF1-AS1 represses p21 expression to promote gastric cancer proliferation through LSD1-Mediated H3K4me2 demethylation [40]. Lnc-DC interacts with STAT3 to induce STAT3 phosphorylation and avoid SHP1-mediated STAT3 dephosphorylation [41]. In triple-negative breast cancer (TNBC), highly-expressed LINK-A actives BRK kinase to phosphorylate HIF-1α and prevent hydroxylation for enhancing p300-HIF-1α association and the resulting HIF-1α transcriptional activity [42]. Here, we provide our finding that LINP1 physically interacts with eIF2α and regulates its phosphorylation. Our finding is in line with the above reports and supports to open a novel unclear territory, where LncRNAs may be “big” players in diversifying the regulatory levels of protein modifications and the following pathophysiologic processes.

Collectively, our study showed LINP1 is higher-expressed and acts as an oncogene in cSCC to promote the proliferation, colony formation, migration and invasiveness of tumor cells. Transcriptomic sequencing and molecular validations confirmed that loss of LINP1 activates UPR signaling and the following apoptosis by inducing PERK/eIF2α signal branch UPR mediator DDIT3 and DR5 expression. Mechanistic study showed that LINP1 interacts with eIF2α to constrain the eIF2α phosphorylation and the induction of DDIT3 for inhibiting UPR signaling-mediated apoptosis, which finally contributes to cSCC progression (Fig. 8G). Our findings highlight the oncogenic and UPR signaling-suppressive roles of LINP1 in cSCC and emphasize a novel regulatory mechanism to constrain UPR signaling and the following apoptosis, which may provide novel intervention targets for designing new therapeutic modality of cSCC.

## Supplementary Information

**Additional file 1: Fig. S1.** Normalized LINP1 expression levels in a variety of tumors analyzed in TCGA database. The original data is from The Cancer Genome Atlas Program (TCGA) database and the diagram shows the expression levels of LINP1 in different types of cancer analyzed by Gene Expression Profiling Interactive Analysis (GEPIA, http://gepia.cancer-pku.cn/ ). **Fig. S2.** LINP1 promotes cell proliferation, migration and invasiveness in HSC-1 cells. (A) LINP1 expression was detected after LINP1 depletion in HSC-1 cells. Measurements of cell proliferation by CCK-8 assay (B), colony formation assay (C), transwell migration assay (D) and Matrigel invasiveness measurement (E) were performed in HSC-1 cells treated with siRNAs targeting LINP1. (F) Apoptosis assay by Annexin V/PI double staining were performed in HSC-1cells treated with siRNAs targeting LINP1. Scale bars, 500 mm (C), 100 μm (D, E). (G) qPCR validations of key gene expression in endoplasmic reticulum signaling including GRP78, XBP1, DDIT3 and DR5 after LINP1 depletion in HSC-1 cells. (H) Protein levels of GRP78, XBP1, eIF2α, p-eIF2α, DDIT3, DR5, and cleavages of Caspase-8, Caspase-7 and Caspase-3 were detected by Western blot after LINP1 knockdown in HSC-1 cells. GAPDH was using as loading control. Each experiment was performed in at least triplicate and results are presented as mean ± s.d. One-way ANOVA and Dunnett’s multiple comparison test were used to analyze the data (*P < 0.05, **P < 0.01, ***P < 0.001). **Fig. S3.** LINP1 promotes cell proliferation, migration and invasiveness in A431 cells. (A, F) LINP1 expression was detected after LINP1 depletion or overexpression in A431 cells. Measurements of cell proliferation by CCK-8 assay (B, G), colony formation assay (C, H), transwell migration assay and Matrigel invasiveness measurement (D, E, I, J) were performed after LINP1 knockdown or overexpression in A431 cells. Scale bars, 500 mm (C, H), 100 μm (D, E, I, J). Each experiment was performed in at least triplicate and results are presented as mean ± s.d. One-way ANOVA and Dunnett’s multiple comparison test were used to analyze the data (*P < 0.05, **P < 0.01, ***P < 0.001). **Fig. S4.** LINP1 functions as oncogene to repress UPR-mediated cell apoptosis in A431 cells. (A) The protein of GRP78, XBP1, eIF2α, p-eIF2α, ATF4 and DDIT3 were detected by Western blot in A431 cells after knockdown of LINP1 or overexpression of LINP1. (B, C) The expression of LINP1, DDIT3, DR5, XBP1 and GRP78 was detected by qRT-PCR in A431 cells after knockdown of LINP1 or overexpression of LINP1. (D) The levels of DR5 and cleaved Caspase-3, cleaved Caspase-7, cleaved Caspase-8 were detected by Western blot in A431 cells after knockdown of LINP1 or overexpression of LINP1. Statistical data of qRT-PCR represent the average of three independent experiments ± s.d. (E, F) Apoptosis assay by Annexin V/PI double staining were performed in LINP1-knockdown or LINP1-overexpression A431 cells. One-way ANOVA and Dunnett’s multiple comparison test were used to analyze the data (*P < 0.05, **P < 0.01, ***P < 0.001). **Fig. S5.** Peptides of eIF2α binding to LINP1 identified by HPLC-MS. (A) The protein mix precipitated by in vitro-transcribed Biotin-labelled LINP1 RNA was analyzed by high performance liquid chromatography-mass spectrometry (HPLC-MS) to identify the amino acid sequences of eIF2α peptides. (B) The ability of eIF2α to interact with LINP1 is predicted by lncPro (http://bioinfo.bjmu.edu.cn/lncpro/). (C) Predicted secondary structure of LINP1 by RNAfold (http://rna.tbi.univie.ac.at//cgi-bin/RNAWebSuite/RNAfold.cgi). **Fig. S6.** Knockdown of DDIT3 promotes the proliferation, migration but represses apoptosis in cSCC cells. (A) DDIT3 expression was detected after depletion of DDIT3 by siNC and siDDIT3 in cSCC cells. (B-E) Depletion of DDIT3 repressed cell apoptosis and enhanced cell proliferation, migration in cSCC cells by CCK-8 assay, colony formation assay, transwell migration assay and Annexin V/PI double staining measurement. Scale bars, 500 mm (C), 100 μm (D). (F) DR5, cleaved forms of Caspase-8, Caspase-7 and Caspase-3 proteins were detected in cSCC cells by Western blot after DDIT3 knockdown, GAPDH was using as loading control. Each experiment was performed in at least triplicate and results are presented as mean ± s.d. One-way ANOVA and Dunnett’s multiple comparison test were used to analyze the data (*P < 0.05, **P < 0.01, ***P < 0.001). **Fig. S7–S9.** (A431, HSC-1 and HaCaT cell lines authentication and quality check).

**Additional file 2: Table S1.** Differentially expressed genes in response to LINP1 depletion

**Additional file 3: Table S2.** KEGG and GO annotation clustering of regulated genes in LINP1-depleted cells

**Additional file 4: Table S3.** List of proteins specifically associating with LINP1 identified by RNA pulldown followed by HPLC-MS

**Additional file 5: Table S4.** KEGG annotation clustering of regulated proteins specifically associating with LINP1 identified by RNA pulldown followed by HPLC-MS

**Additional file 6: Table S5.** Oligonucleotides used for qRT-PCR, knockout, knockdown and ISH detection

## Data Availability

RNA-seq data have been deposited in NCBI GEO datasets with the following accession codes: GSE197844. The mass spectrometry proteomics data have been deposited to the ProteomeXchange [47] (http://www.proteomexchange.org) Consortium via the PRIDE [48] partner repository with the dataset identifier PXD032027. All data generated or analysed during this study are included in this published article and its expanded view files and available from the corresponding author on request.

## Abbreviations

ER: Endoplasmic reticulum
UPR: Unfolded protein response
cSCC: Cutaneous squamous cell carcinoma
LINP1: LncRNA in non-homologous end joining pathway 1
TM: Tunicamycin
NHEK: Primary normal human epidermal keratinocytes
TCGA: The Cancer Genome Atlas
TNBC: Triple-negative breast cancer
NHEJ: Non-homologous end joining
KEGG: The Kyoto Encyclopedia of Genes and Genome
DAVID: The database for annotation, visualization, and integrated discovery
GO: Ontology
ThT: Thioflavin T
