# Supplementary material for: LINP1 represses unfolded protein response by directly inhibiting eIF2α phosphorylation to promote cutaneous squamous cell carcinoma
Source: Exp Hematol Oncol. 2023 Mar 14;12:31. doi: 10.1186/s40164-023-00395-1 (PMC10012465; doi:10.1186/s40164-023-00395-1)
Supplement: Supplementary file 1 — Additional file 1: Fig. S1. Normalized LINP1 expression levels in a variety of tumors analyzed in TCGA database. The original data is from The Cancer Genome Atlas Program (TCGA) database and the diagram shows the expression levels of LINP1 in different types of cancer analyzed by Gene Expression Profiling Interactive Analysis (GEPIA, http://gepia.cancer-pku.cn/ ). Fig. S2. LINP1 promotes cell proliferation, migration and invasiveness in HSC-1 cells. (A) LINP1 expression was detected after LINP1 depletion in HSC-1 cells. Measurements of cell proliferation by CCK-8 assay (B), colony formation assay (C), transwell migration assay (D) and Matrigel invasiveness measurement (E) were performed in HSC-1 cells treated with siRNAs targeting LINP1. (F) Apoptosis assay by Annexin V/PI double staining were performed in HSC-1cells treated with siRNAs targeting LINP1. Scale bars, 500 mm (C), 100 μm (D, E). (G) qPCR validations of key gene expression in endoplasmic reticulum signaling including GRP78, XBP1, DDIT3 and DR5 after LINP1 depletion in HSC-1 cells. (H) Protein levels of GRP78, XBP1, eIF2α, p-eIF2α, DDIT3, DR5, and cleavages of Caspase-8, Caspase-7 and Caspase-3 were detected by Western blot after LINP1 knockdown in HSC-1 cells. GAPDH was using as loading control. Each experiment was performed in at least triplicate and results are presented as mean ± s.d. One-way ANOVA and Dunnett’s multiple comparison test were used to analyze the data (*P < 0.05, **P < 0.01, ***P < 0.001). Fig. S3. LINP1 promotes cell proliferation, migration and invasiveness in A431 cells. (A, F) LINP1 expression was detected after LINP1 depletion or overexpression in A431 cells. Measurements of cell proliferation by CCK-8 assay (B, G), colony formation assay (C, H), transwell migration assay and Matrigel invasiveness measurement (D, E, I, J) were performed after LINP1 knockdown or overexpression in A431 cells. Scale bars, 500 mm (C, H), 100 μm (D, E, I, J). Each experiment was performed in at least tr [file 40164_2023_395_MOESM1_ESM.pdf]

Additional file 1: Figure S1

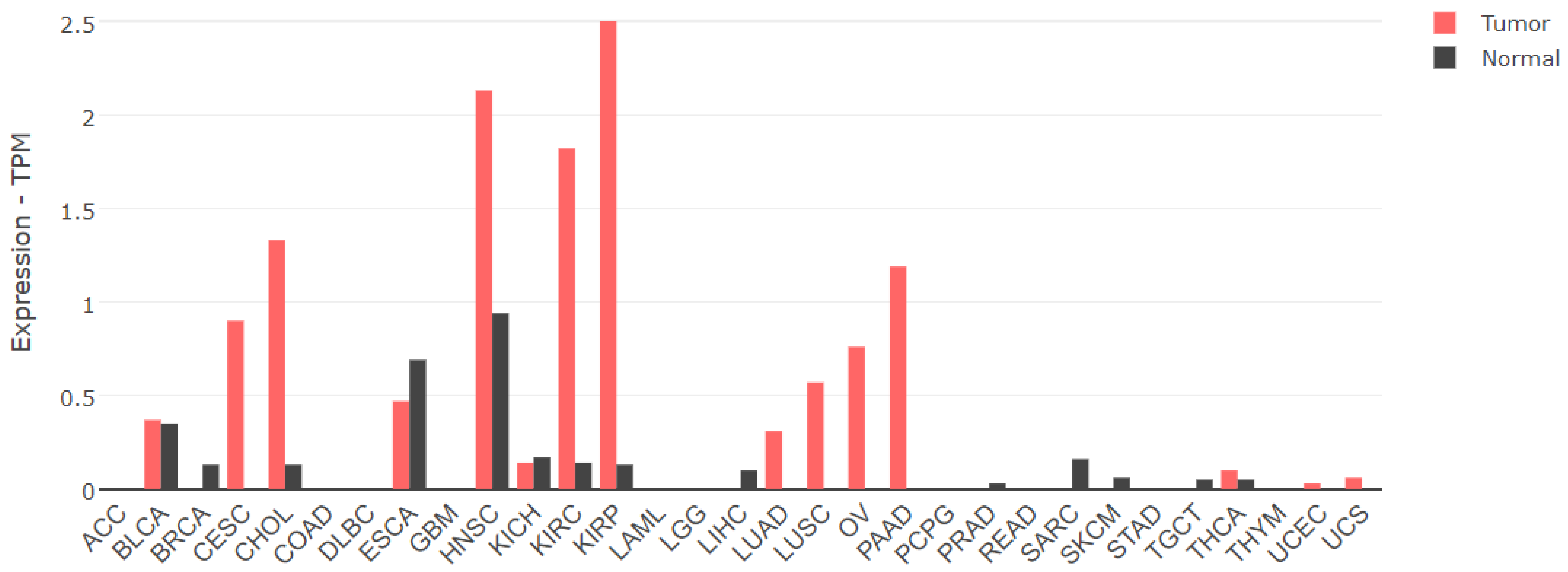

Additional file 1:Figure S1. Normalized LINP1 expression levels in a variety of tumors analyzed in TCGA database. The original data is from The Cancer Genome Atlas Program (TCGA) database and the diagram showing the expression levels of LINP1 in different types of cancer analyzed by Gene Expression Profiling Interactive Analysis (GEPIA, http://gepia.cancer-pku.cn/).

Additional file 1: Figure S2

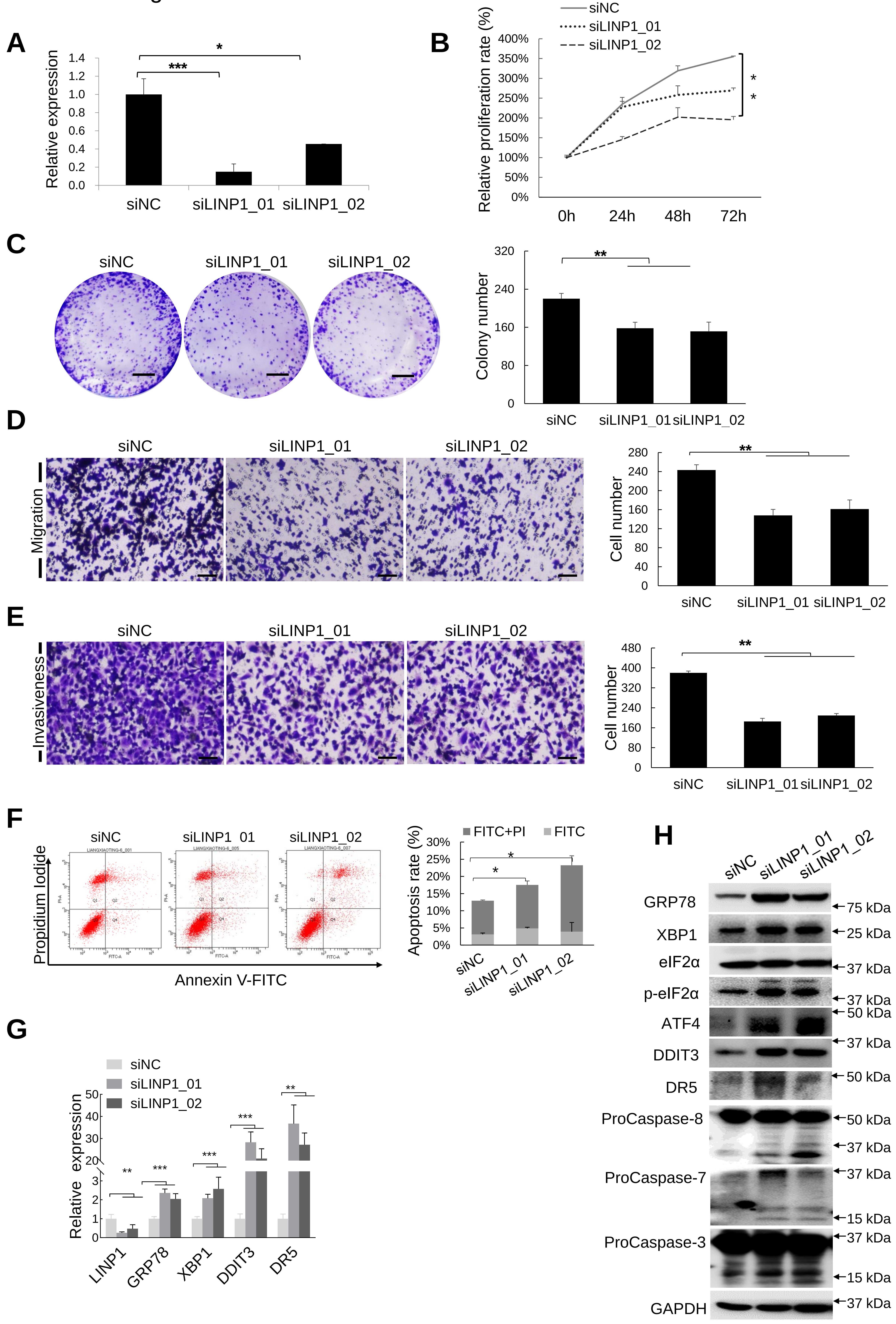

Additional file 1: Figure S2

Affitional file 1: Figure S2. **LINP1 promotes cell proliferation, migration and invasiveness in HSC-1 cells.** (A) LINP1 expression was detected after LINP1 depletion in HSC-1 cells. Measurements of cell proliferation by CCK-8 assay (B), colony formation assay (C), transwell migration assay (D) and Matrigel invasiveness measurement (E) were performed in HSC-1 cells treated with siRNAs targeting LINP1. (F) Apoptosis assay by Annexin V/PI double staining were performed in HSC-1cells treated with siRNAs targeting LINP1. Scale bars, 500 mm (C), 100 μm (D,E). (G) qPCR validations of key gene expression in endoplasmic reticulum signaling including GRP78, XBP1, DDIT3 and DR5 after LINP1 depletion in HSC-1 cells. (H) Protein levels of GRP78, XBP1, eIF2α, p-eIF2α, DDIT3, DR5, and cleavages of Caspase-8, Caspase-7 and Caspase-3 were detected by Western blot after LINP1 knockdown in HSC-1 cells. GAPDH was using as loading control. Each experiment was performed in at least triplicate and results are presented as mean ± s.d. One-way ANOVA and Dunnett's multiple comparison test were used to analyze the data (*$P < 0.05$, **$P < 0.01$, ***$P < 0.001$).

Additional file 1: Figure S3

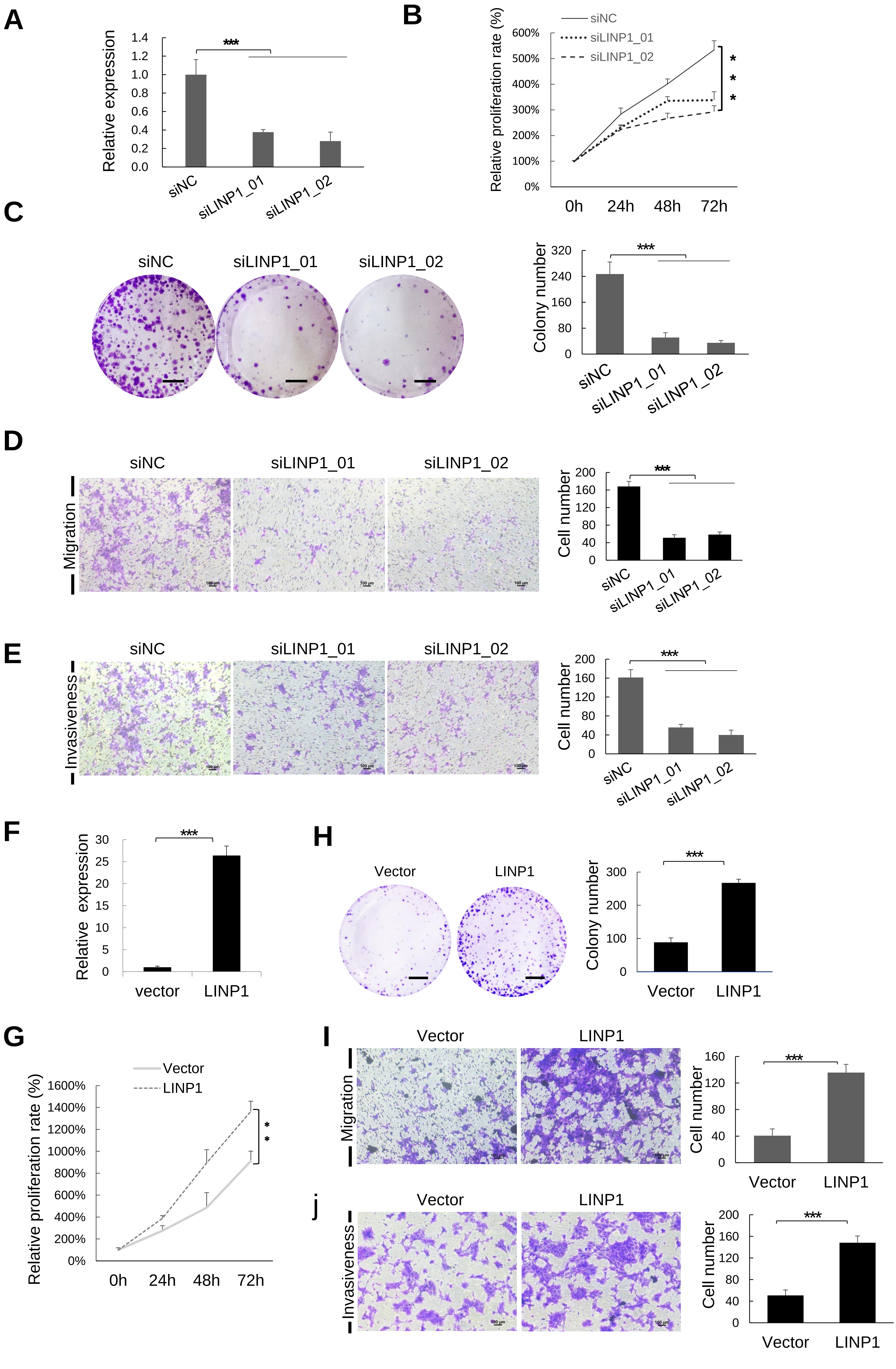

Additional file 1: Figure S3. **LINP1 promotes cell proliferation, migration and invasiveness in A431 cells.** (A, F) LINP1 expression was detected after LINP1 depletion or overexpression in A431 cells. Measurements of cell proliferation by CCK-8 assay (B, G), colony formation assay (C, H), transwell migration assay and Matrigel invasiveness measurement (D, E, I, J) were performed in A431 cells treated with siRNAs targeting LINP1 or overexpressing LINP1. Scale bars, 500 mm (C, H), 100 μm (D, E, I, J). Each experiment was performed in at least triplicate and results are presented as mean ± s.d. One-way ANOVA and Dunnett's multiple comparison test were used to analyze the data (**P* < 0.05, ***P* < 0.01, ****P* < 0.001).

Additional file 1: Figure S4

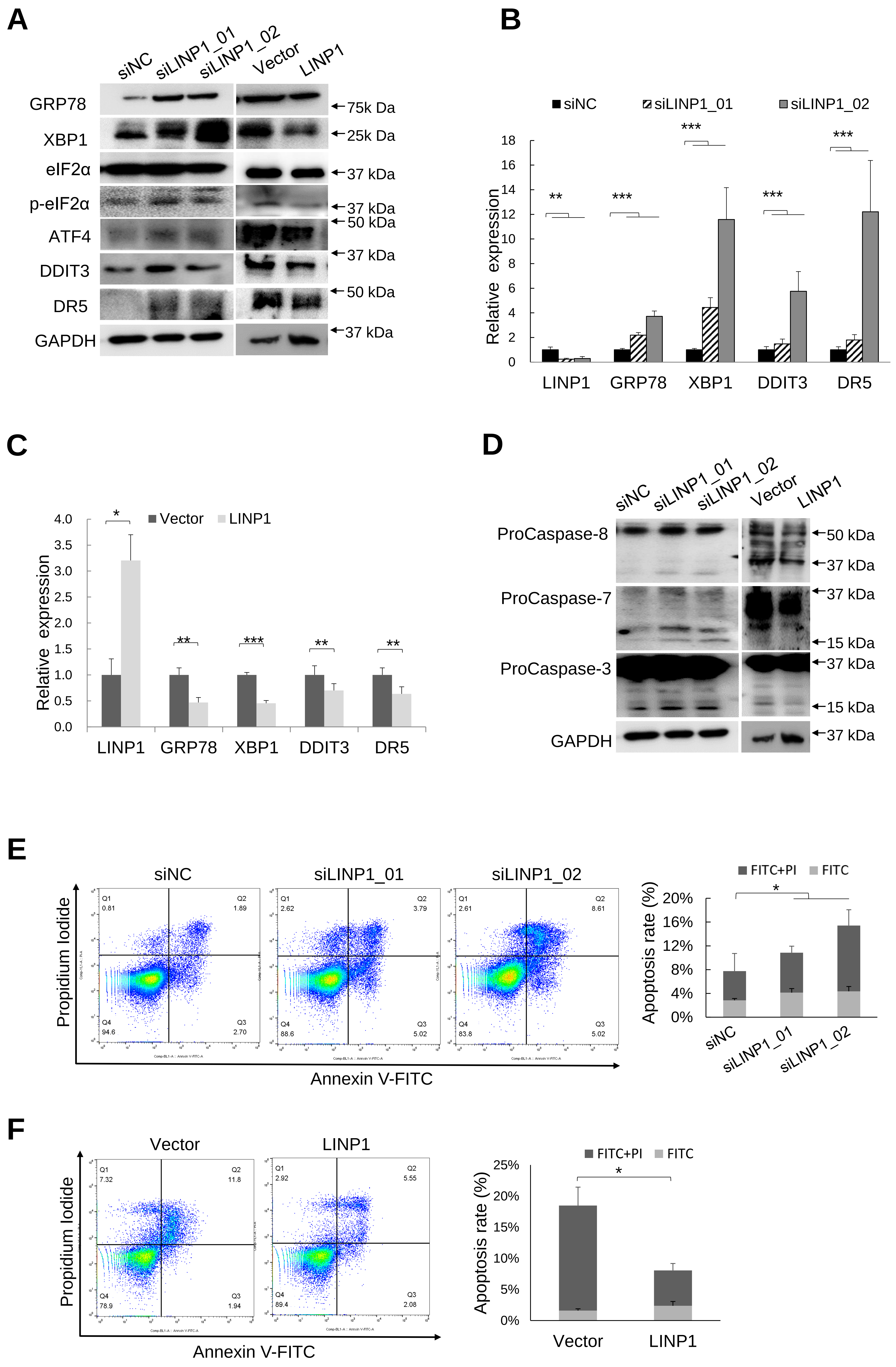

Additional file 1:Figure S4. LINP1 functions as oncogene to repress UPR-mediated cell apoptosis in A431 cells. (A) The protein of GRP78, XBP1, eIF2α, p-eIF2α, ATF4 and DDIT3 were detected by Western blot in A431 cells after knockdown of LINP1 or overexpression of LINP1. (B,C) The expression of LINP1, DDIT3, DR5, XBP1 and GRP78 was detected by qRT-PCR in A431 cells after knockdown of LINP1 or overexpression of LINP1. (D) The levels of DR5 and cleaved Caspase-3, cleaved Caspase-7, cleaved Caspase-8 were detected by Western blot in A431 cells after knockdown of LINP1 or overexpression of LINP1. Statistical data of qRT-PCR represent the average of three independent experiments ± s.d. (E, F) Apoptosis assay by Annexin V/PI double staining were performed in A431 cells treated with siRNAs targeting LINP1 or overexpressing LINP1. One-way ANOVA and Dunnett's multiple comparison test were used to analyze the data (*$P < 0.05$, **$P < 0.01$, ***$P < 0.001$).

Additional file 1: Figure S5

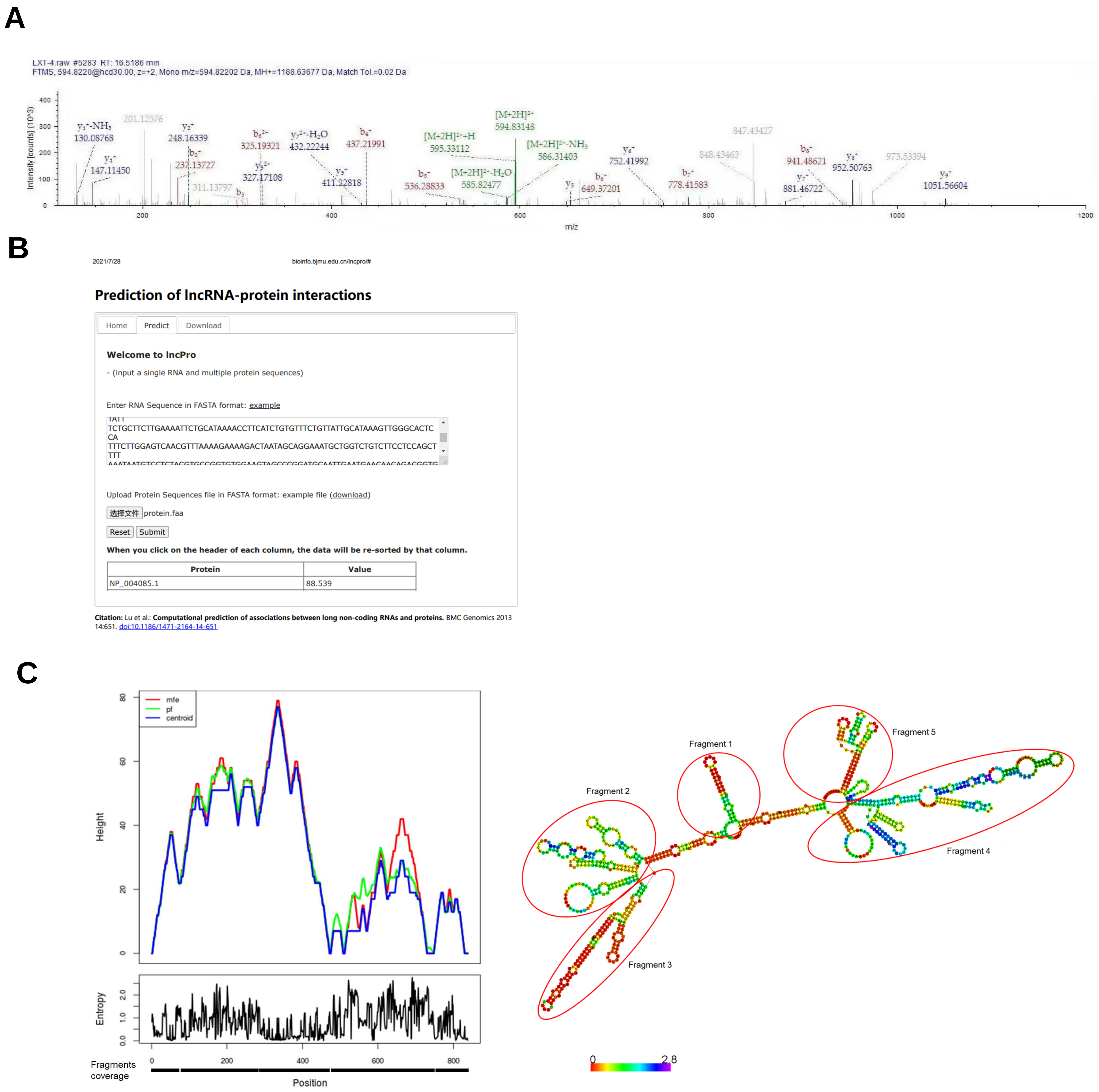

Additional file 1:Figure S5. Peptides of eIF2α binding to LINP1 identified by HPLC-MS. (A) The protein mix precipitated by *in vitro*-transcribed Biotin-labelled LINP1 RNA was analyzed by high performance liquid chromatography-mass spectrometry (HPLC-MS) to identify the amino acid sequences of eIF2α peptides. (B) The ability of eIF2α to interact with LINP1 is predicted by lncPro (http://bioinfo.bjmu.edu.cn/lncpro/). (C) Predicted secondary structure of LINP1 by RNAfold (http://rna.tbi.univie.ac.at//cgi-bin/RNAWebSuite/RNAfold.cgi).

Additional file 1: Figure S6

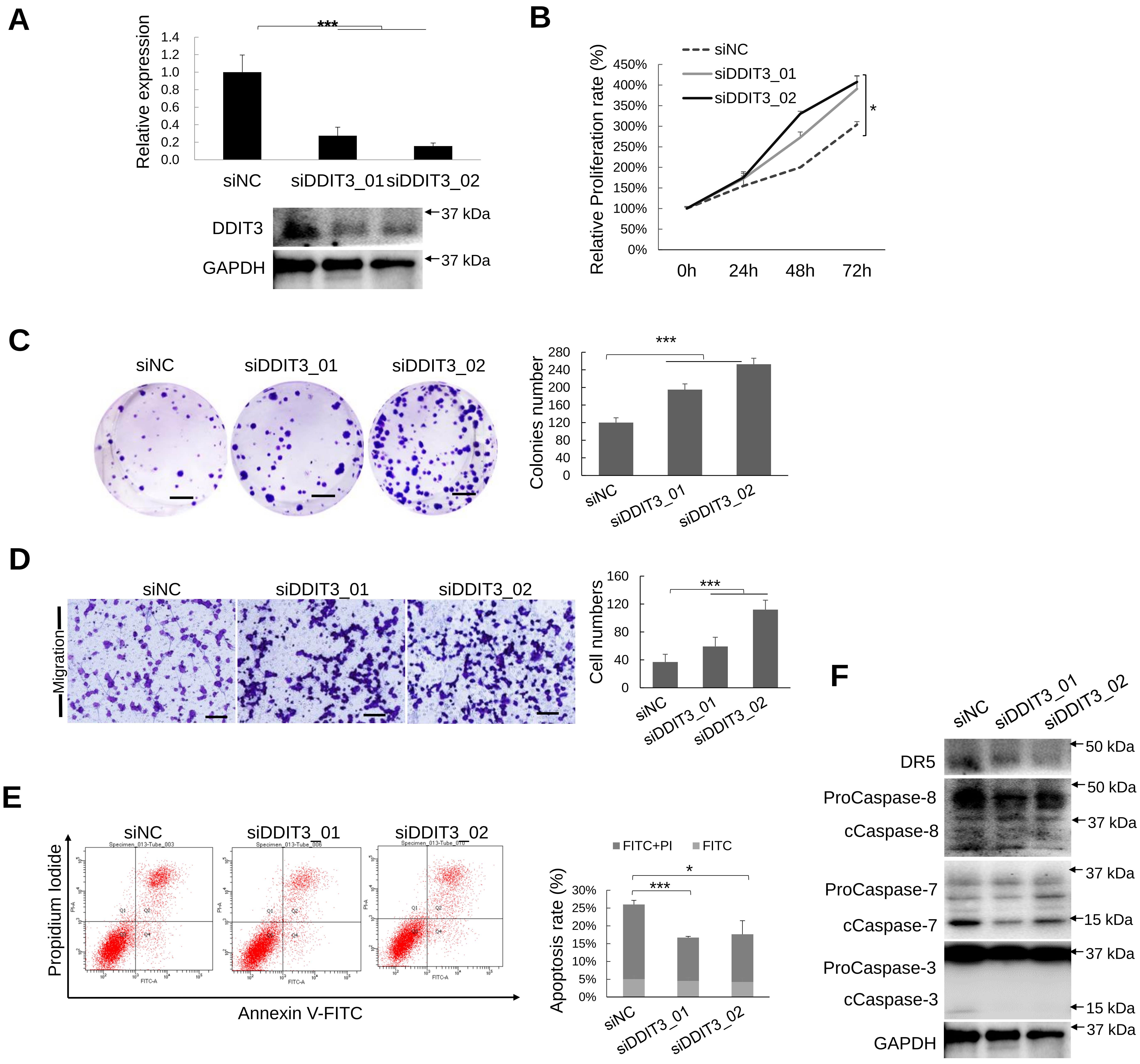

Additional file 1: Figure S6. Knockdown of DDIT3 promotes the proliferation, migration but represses apoptosis in cSCC cells. (A) DDIT3 expression was detected after depletion of DDIT3 by siNC and siDDIT3 in cSCC cells. (B-E) Depletion of DDIT3 repressed cell apoptosis and enhanced cell proliferation, migration in cSCC cells by CCK-8 assay, colony formation assay, transwell migration assay and Annexin V/PI double staining measurement. Scale bars, 500 mm (C), 100 μm (D). (F) DR5, cleaved forms of Caspase-8, Caspase-7 and Caspase-3 proteins were detected in cSCC cells by Western blot after DDIT3 knockdown, GAPDH was using as loading control. Each experiment was performed in at least triplicate and results are presented as mean ± s.d. One-way ANOVA and Dunnett's multiple comparison test were used to analyze the data (*$P < 0.05$, **$P < 0.01$, ***$P < 0.001$).

Additional file 1: Figure S7

A431 cell line authentication and quality check

A431 细胞 STR 鉴定报告

一、 材料处理和检验方法

取适量 **A431** 细胞(1×10^6)使用 PureLink® Genomic DNA Mini Kit（美国 Life K182001）提前基因组 DNA，采用 PowerPlex®18D 系统（美国 Promega DC1802）试剂盒进行扩增，在 ABI3500 Genetic Analyzer（美国 Life3500）进行检测。

二、 检测结果

实验中阴性及阳性对照结果均正确。

A431 细胞株的 STR 位点和 Amelogenin 位点的基因分型结果见附表，分型图谱见附图。

三、 分析说明

A431 细胞株基因组 DNA 扩增后图谱清晰，分型结果良好。

四、 检验结论

1. **A431** 细胞株 DNA 进行细胞 STR 分型结果显示，细胞株中未发现人类细胞交叉污染。
2. 该细胞株 DNA 分型在 ATCC 细胞库中找到与其细胞分型 100%相匹配的细胞株，细胞株名称为 **A431**。

A-431 细胞出库质检单

一、产品信息

1. 细胞名称：A-431（人表皮癌细胞）
2. 细胞货号：CL-0015
3. 出库日期：2019-12-27

二、检测项目及结果

检测项目	检测结果	
生长特性（贴壁/悬浮）	贴壁	
细胞形态	上皮细胞样	
细胞密度	＞75%	
细胞纯度	——	
细胞总量	~1×10^6/Cells	
细胞活力	＞95%	
HIV-1	有□	无☑
HBV	有□	无☑
HCV	有□	无☑
支原体	有□	无☑
细菌	有□	无☑
酵母	有□	无☑
真菌	有□	无☑

三、质检员及质检日期

质检员：王帅　　质检日期：2019-12-24

质检专用章

质量合格，准予放行

网站：www.procell.com.cn

Procell Life Science&Technology Co,.Ltd.

附表 1：细胞株 A431 的 STR 位点和 Amelogenin 位点的基因分型结果

细胞 **A431**（图片编号为 PC79）		
**Marker**	**Allele 1**	**Allele 2**
D3S1358	14	14
TH01	9	9
D21S11	28	30
D18S51	13	17
Penta E	12	13
D5S818	12	13
D13S317	9	13
D7S820	10	10
D16S539	12	14
CSF1PO	11	12
Penta D	11	11
AMEL	X	X
vWA	15	17
D8S1179	13	13
TPOX	11	11
FGA	20	20
D6S1043	18	18
D2S1338	17	20
D12S391	18	23
D19S433	15	15.2
D1S1656	16	16

附图 1：ATCC 官网 A431 细胞 STR 位点信息

A-431 (ATCC® CRL-1555™)

Organism: **Homo sapiens, human** / Tissue: **skin/epidermis** / Disease: **epidermoi**

GENERAL INFORMATION | CHARACTERISTICS | CULTURE METHOD | SPECIFICATIONS

STR Profile

Amelogenin: X
CSF1PO: 11,12
D13S317: 9,13
D16S539: 12,14
D5S818: 12,13
D7S820: 10

附图 2：A431 细胞 STR 位点和 Amelogenin 位点的基因分型结果

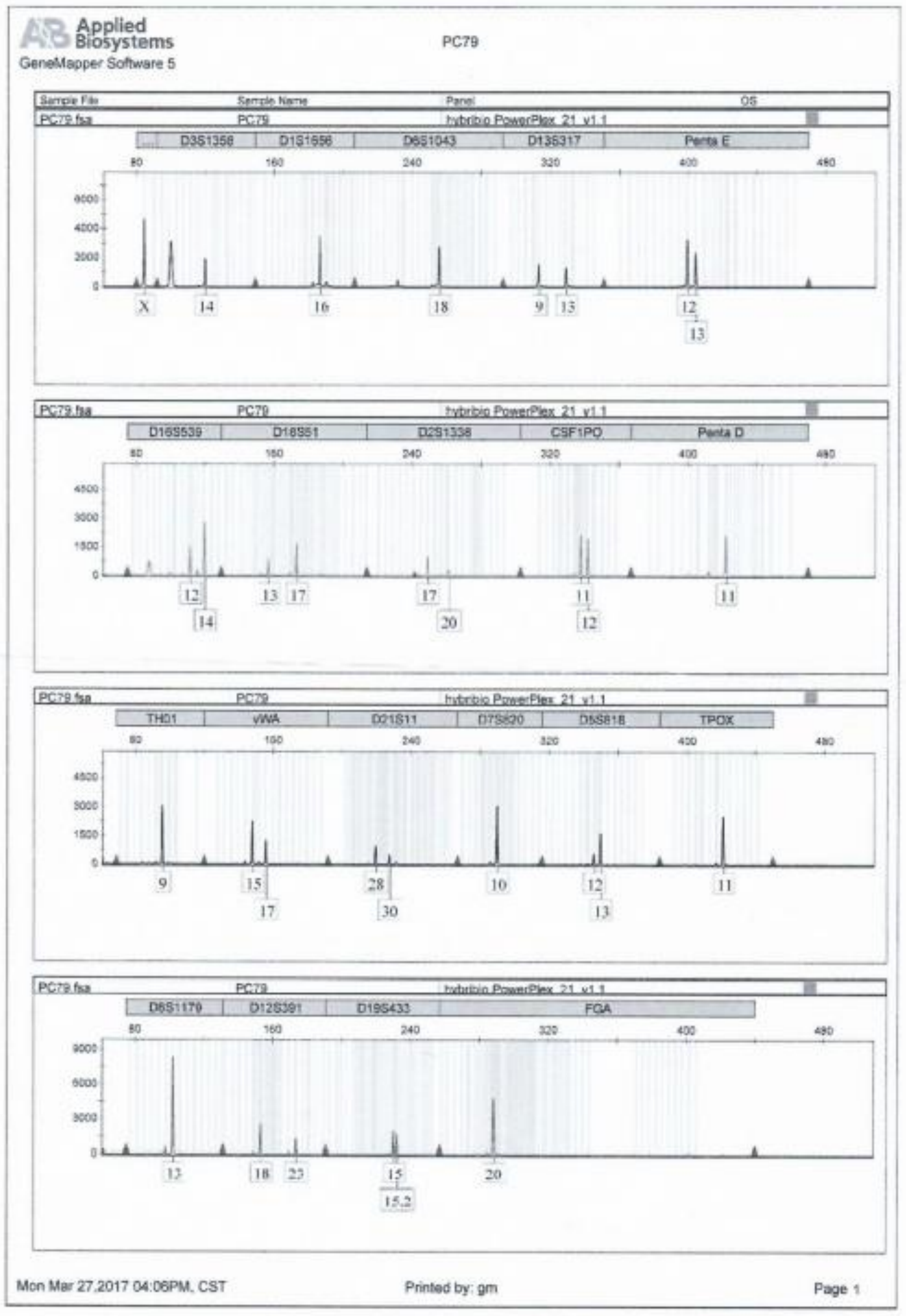

Additional file 1: Figure S8

HSC-1 cell line authentication and quality check

样品编号:

表 1 样本编号

客户样本编号	公司编号
HSC-1	20170206-01

样品数量: 1

样品性状: 细胞系

检测项目: STR

送检单位: 弘顺生物

检测方法: 用 Axygen 的基因组抽提试剂盒提取 DNA，采用 20- STR 扩增方案扩增，在 ABI 3730XL 型遗传分析仪上对 STR 位点和性别基因 Amelogenin 进行检测。

(一)检验基本情况

表 2：样本基因型检验结果

样本编号	多等位基因	匹配细胞系	细胞库	EV 值	匹配说明
20170206-01	无	HSC-1	JCRB	1	完全匹配

- 多等位基因指三等位及以上基因现象。
- 本次检测各细胞分型结果良好。

(二)各样本描述

- 20170206-01：该株细胞 DNA 分型在细胞系检索中找到**完全匹配**的细胞系，JCRB 数据库显示细胞名为 **HSC-1**，细胞号对应 **JCRB1015**。本次检测在该细胞系中**没有发现多等位基因**。
- **备注：**待测细胞系与收录于 ATCC, DSMZ, JCRB 和 RIKEN 数据库的细胞系 STR 数据进行比对，未收录于以上细胞库的细胞系将无法匹配。

(三)样本分型结果

表 3：细胞 20170206-01 的 STR 位点和 Amelogenin 位点的基因分型结果

Marker	样本				细胞库信息		
	Allele1	Allele2	Allele3	Allele4	Allele1	Allele2	Allele3
D5S818	10	13			10	13	
D13S317	11	12			11	12	
D7S820	11	12			11	12	
D16S539	11	12			11	12	
VWA	16	17			16	17	
TH01	7	7			7	7	
AMEL	X	Y			X	Y	
TPOX	8	8			8	8	
CSF1PO	12	13			12	13	
D12S391	20	25					
FGA	20	20					
D2S1338	19	19					
D21S11	30	30					
D18S51	21	21					
D8S1179	12	14					
D3S1358	16	18					
D6S1043	18	18					
PENTAE	15	17					
D19S433	12.2	13					
PENTAD	9	9					

其他说明:

(一)分型方案及位点分布:

附表：实验方案及位点

	方案 1	方案 2	方案 3	方案 4	方案 5	方案 6
1	TH01	AMEL	TPOX	D3S1358	PENTAE	D19S433
2	D12S391	D5S818	VWA	D13S317		PENTAD
3	D7S820	D2S1338	D8S1179	D6S1043		
4	CSF1PO	D21S11		D16S539		
5	FGA	D18S51				

(二)STR 数据库比对:

本公司采用 DSMZ tools 进行细胞系比对，其中包含来自于 ATCC, DSMZ, JCRB 和 RIKEN 数据库的 2455 个细胞系 STR 数据。如果待检测细胞未收录于以上细胞库或这是自行建立的新细胞系将无法进行比对，用户需根据细胞分型结果自行与其他数据库进行比对。

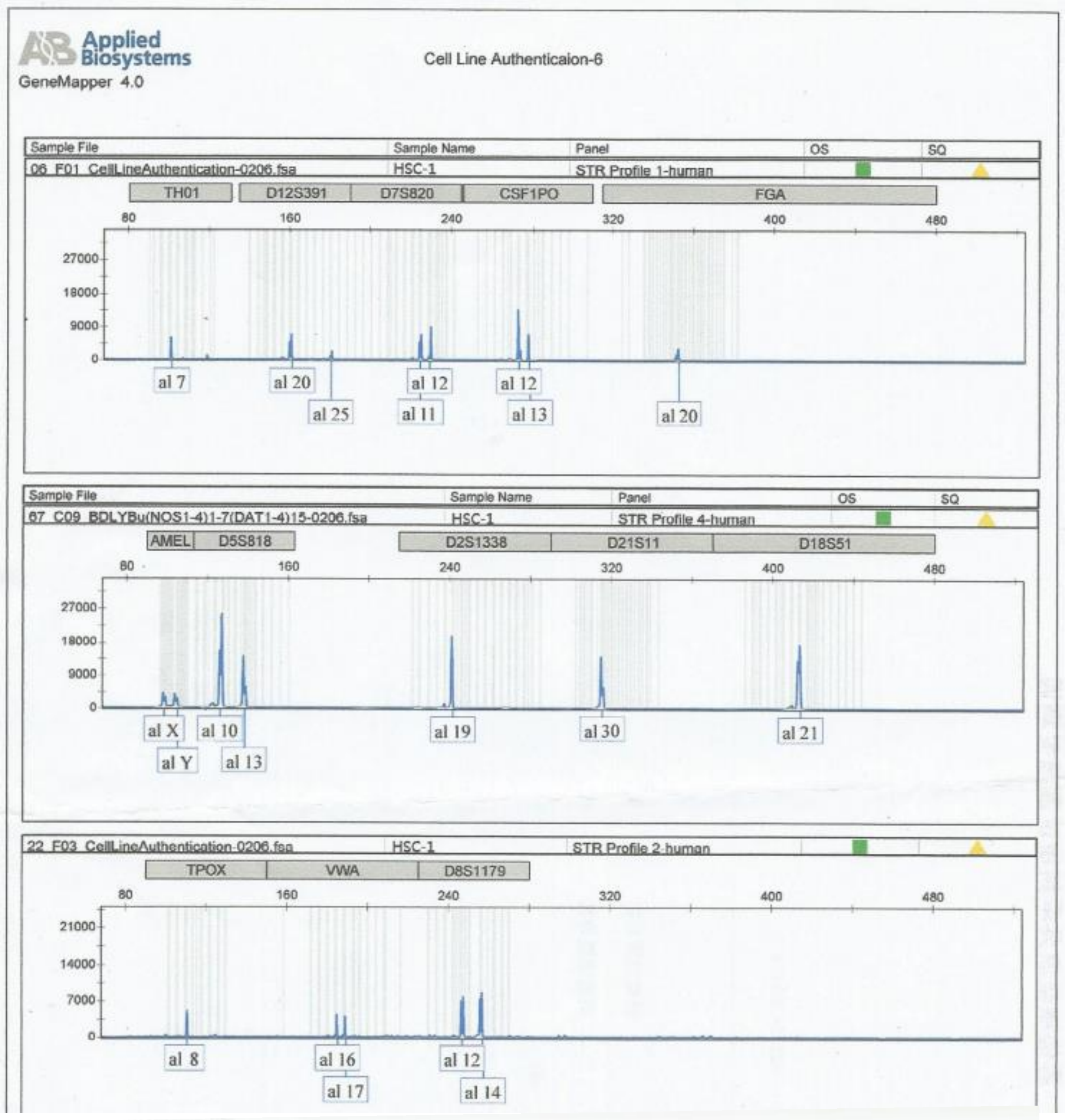

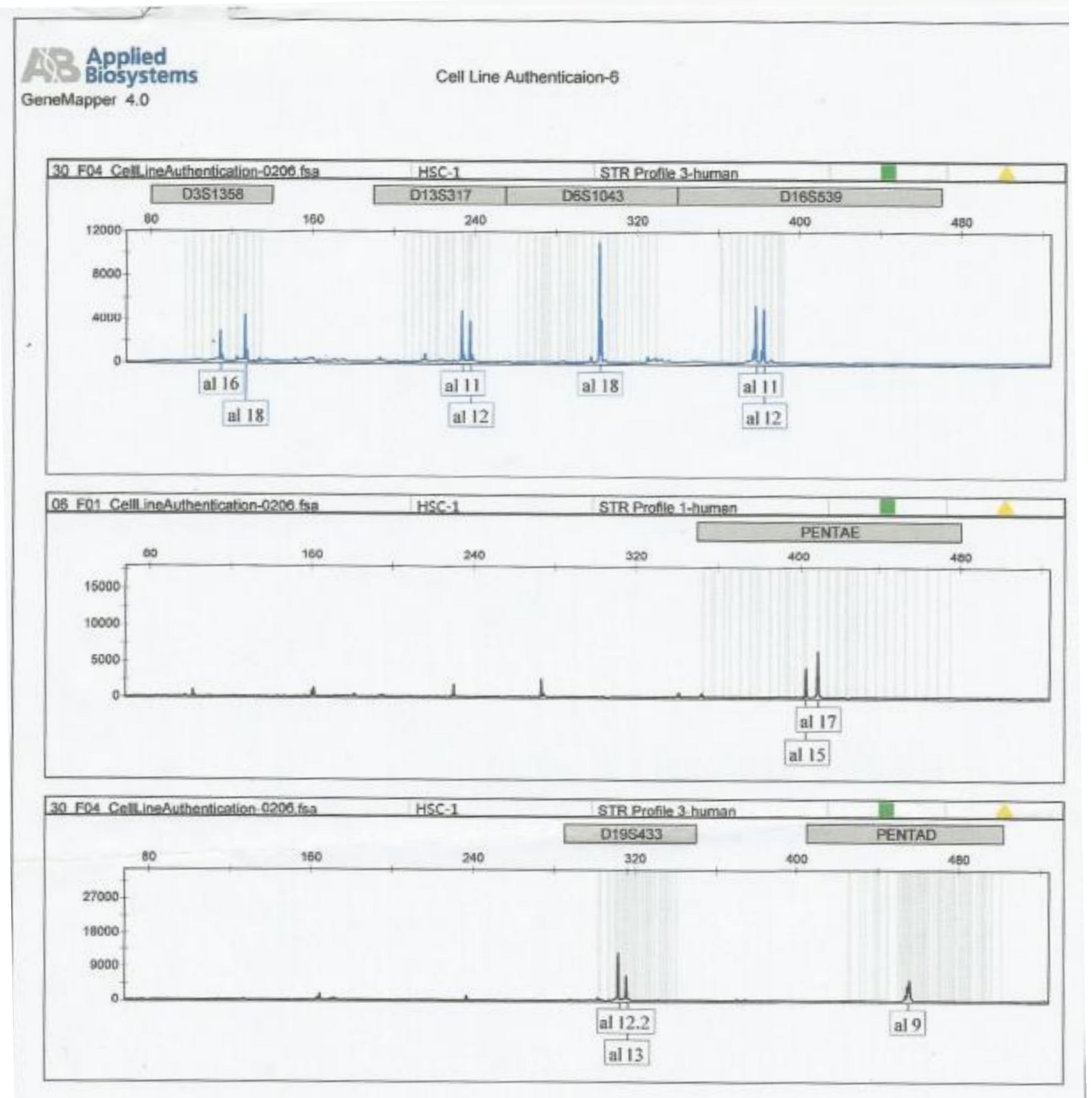

Additional file 1: Figure S9

HaCaT cell line authentication and quality check

Report of Human Cell Line Authentication

(Notice:This authentication report is restricted to the cell sold from Guangzhou Cellcook Biotech Co., Ltd, and the date with seal is the date of delivery.)

Ⅰ. Sample

Sample Name: labeled as 'HaCaT'

Ⅱ. Method and Procedure

1. PCR is amplified with STR Multi-amplification Kit (PowerPlex™16HS System);
2. PCR products are assayed with 3100 DNA Analyzer (Applied Biosystems®).
3. Amplification of gene COX1 and electrophoresis are employed to survey the species of the sample.

Ⅲ. Results

1. The STR profiles of the cell line sample are in the attached table and figure.
2. The search result in ATCC and DSMZ databases.
3. The electrophoresis figure of gene COX1.

HaCaT: ①Two loci have tri-alleles(D5S818 and CSF1P0). Contamination of other human cell line is not found (Figure 1 & Table 1). ②100% matched cell lines are not found in ATCC and DSMZ data banks (Figure 2 & Figure 3).③The sample is a human cell line. Contamination of other species cells are not found in the sample (Figure 4).

Operator: Xiaohua Mo Auditor: Xuanyi Liang

GUANGZHOU CELLCOOK BIOTECH CO.,LTD 15 OCT 2018 CELL STR PROFILING PASS

Guangzhou Cellcook Biotech Co., Ltd

Catalog No.: CC4013
Cell Name: HaCaT
Size: T25 culture flask, 1×10^6cells
Morphology: Epithelial
Culture Properties: Adherent
Characteristics: The cell line was derived from the normal skin around the lesion of a 62 years old male patient with melanoma. Keratin and filaggrin are positive.
Culture Method: MEM 10%FBS
Subcultivation Ratio: 1:10-1:20; Twice per week
Trypsined Time: 3-5 minutes
STR Profile:

STR Profile	AMEL	CSF1PO	D13S317	D16S539	D5S818	D7S820	THO1	TPOX	vWA
HaCaT	X	9 11	10 12	9 12	12	9 11	9. 3	11 12	16 17

Figure 1. STR profiles of HaCaT cell line

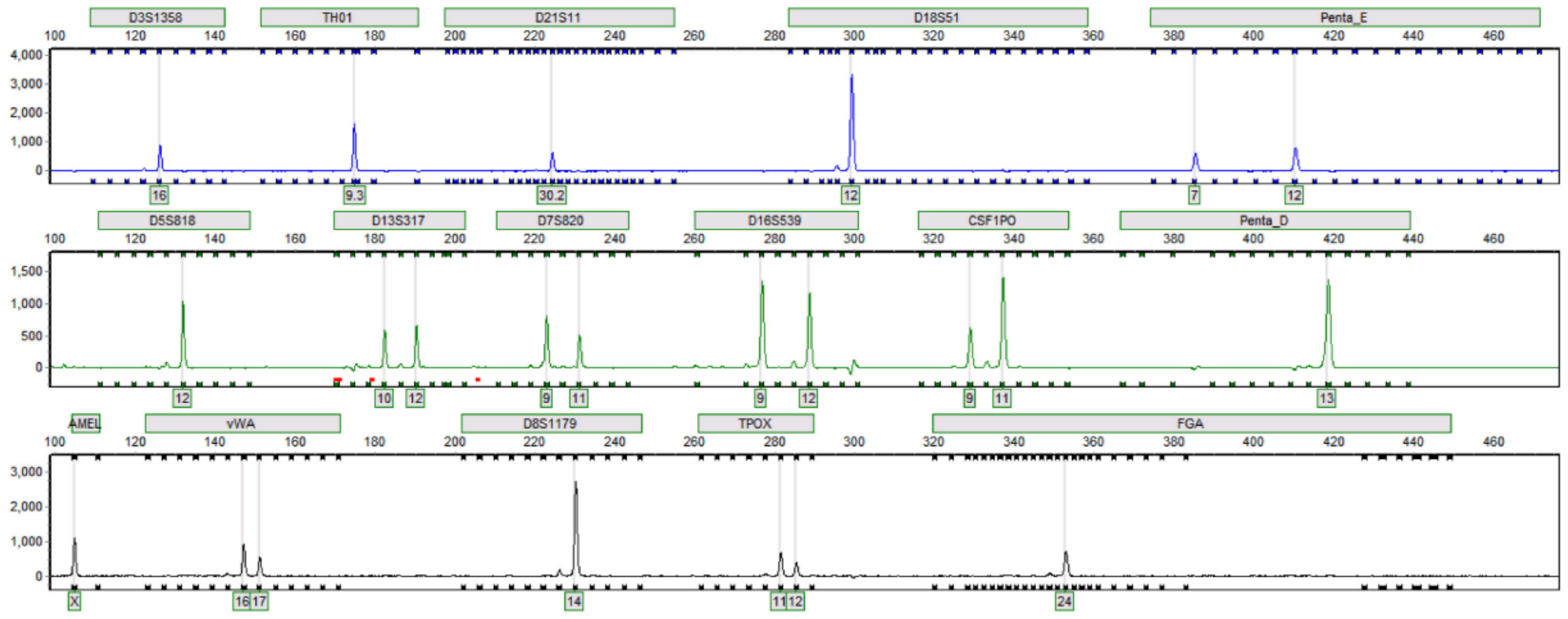

Table 1. STR profiles of HaCaT cell line

	Allele1	Allele2
D3S1358	16	
TH01	9. 3	
D21S11	30. 2	
D18S51	12	
Penta_E	7	12
D5S818	12	
D13S317	10	12
D7S820	9	11
D16S539	9	12
CSF1P0	9	11
Penta_D	13	
AMEL	x	
vWA	16	17
D8S1179	14	
TPOX	11	12
FGA	24	

Figure 2. Search result in ATCC database

SEARCH THE STR DATABASE

As part of our continuing efforts to characterize and authenticate the cell lines in the Cell Biology collection, ATCC has developed a comprehensive database of short tandem repeat (STR) DNA profiles for all of our human cell lines. View our brief tutorial before starting.

1. STR Profiling Analysis
2. Matching Algorithm
3. Interrogating the Database

Showing 1 - 1 Of 1 PageSize: 100

Add to Cart	%Match	ATCC® Number	Designation	D5S818	D13S317	D7S820	D16S539	vWA	TH01	AMEL	TPOX	CSF1PO
	91.0	CRL-3298	MP46Uveal MelanomaHuman	12	10,12	11	12	16,17	9	X	11	11

Figure 3. Search result in DSMZ database

Result of STR matching analysis by your data.

- DSMZ Profile Database -

A graphical presentation is shown at the bottom of this page.

EV	Cell No.	Cell name	Locus names									Figures
			D5S818	D13S317	D7S820	D16S539	VWA	TH01	AM	TPOX	CSF1PO	
	Query (Your Cell)		*12*	*10,12*	*9,11*	*9,12*	*16,17*	*9.3*	*x*	*11,12*	*9,11*	
1.09(36/33)	771	HACAT	12,12	10,12	9,11	9,12	16,17	9.3,9.3	X,X	11,12	9,11	-
0.85(28/33)	704	OAC-P4C	9,9	12,12	9,11	12,12	16,16	9,9	X,X	11,11	11,11	-
0.85(28/33)	CRL-1611	ACHN	12,12	12,12	9,11	12,13	16,17	8,8	X,X	8,11	11,11	-
0.85(28/33)	CRL-7826	Hs 38.T	11,11	12,12	9,9	9,9	17,17	9,9	X,X	11,11	11,11	-
0.85(28/33)	RCB1962	ACHN	12,12	12,12	9,11	12,13	16,17	8,8	X,X	8,11	11,11	-
0.79(26/33)	15	ML-2	12,12	9,12	9,11	9,12	16,16	7,9.3	X,X	8,10	10,11	-
0.79(26/33)	432	8-MG-BA	10,12	12,12	11,11	12,12	17,17	9,9.3	X,X	8,10	10,11	-
0.79(26/33)	661	UPCI-SCC-172	12,12	11,11	9,11	10,10	17,18	9.3,9.3	X,X	11,11	11,11	-
0.79(26/33)	CRL-10442	HCN-1A	11,12	11,12	11,12	12,12	17,17	9.3,9.3	X,X	11,11	10,10	-
0.79(26/33)	CRL-1593.2	U-937	12,12	10,12	9,11	12,12	15,15	9.3,9.3	X,X	8,11	12,12	-
0.79(26/33)	CRL-1855	LCL 721.221	12,12	11,12	10,11	12,12	14,17	9.3,9.3	X,X	8,12	11,12	-
0.79(26/33)	CRL-2367	TUR	12,12	10,12	9,11	12,12	14,16	6,9.3	X,X	8,11	12,12	-

Figure 4. Authentication of the species of the sample

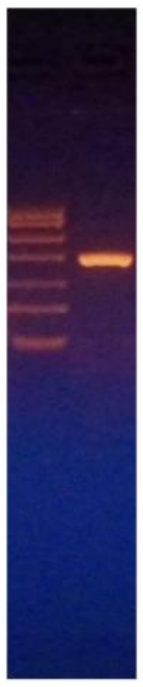

M: Marker. As the size of 700, 600, 500, 400, 300, 200 and 100bp from up to down.

Nine species are checked, as follow: *Homo sapiens* 391bp, *Cricetulus griseus*315bp, *Macaca mulatta*287bp, *Cercopithecus aethiops*222bp, *Rattus norvegicus*196bp, *Canis familiaris*172bp, *Mus musculus*150bp, *Bos Taurus*102bp, IC 70bp

The sample: The band size is 391bp which matches the size of human.
